# Nanoparticle-Based Therapies for Myocardial Injury and Heart Failure: A Systematic Review and Translational Appraisal of Preclinical Evidence

**DOI:** 10.3390/biom16091245

**Published:** 2026-08-27

**Authors:** Ayesha Jabeen, Ilaria Barison, Honoria Ocagli, Bruna Fata, Diego Perazzolo, Cristina Basso, Roberto Luisetto, Silvia Pozzo, Fabrizio Mancin, Enrico Grisan, Dario Gregori, Annalisa Angelini, Marny Fedrigo, Chiara Castellani

**Affiliations:** 1 Department of Cardiac, Thoracic, Vascular Sciences and Public Health, University of Padua, 35121 Padua, Italy; ayesha.jabeen@studenti.unipd.it (A.J.); ilaria.barison.1@studenti.unipd.it (I.B.); bruna.fata@studenti.unipd.it (B.F.); diego.perazzolo.1@studenti.unipd.it (D.P.); 2 Unit of Biostatistics, Epidemiology and Public Health, Department of Cardiac, Thoracic, Vascular Sciences and Public Health, University of Padua, 35121 Padua, Italy; honoria.ocagli@unipd.it (H.O.); dario.gregori@ubep.unipd.it (D.G.); 3 BIOSTAT-X Biostatistics & AI for Biomedical Discovery, Pediatric Research Institute (IRP) “Città Della Speranza”, 35121 Padua, Italy; 4 Cardiovascular Pathology Unit, University Hospital of Padua, Department of Cardiac, Thoracic, Vascular Sciences and Public Health, University of Padua, 35121 Padua, Italy; cristina.basso@unipd.it (C.B.); annalisa.angelini@unipd.it (A.A.); 5 Department of Surgical, Oncological and Gastroenterological Sciences, University of Padua, 35121 Padua, Italy; roberto.luisetto@unipd.it; 6 Department of Chemical Sciences, University of Padua, 35121 Padua, Italy; silvia.pozzo.1@studenti.unipd.it (S.P.); fabrizio.mancin@unipd.it (F.M.); 7 School of Computer Science and Digital Technologies, REACT Innovation Center, London South Bank University, London CR0 1LB, UK; enrico.grisan@lsbu.ac.uk; 8 Cardiovascular Pathology Unit, Department of Integrated Diagnostic Service, University Hospital of Padua, 35121 Padua, Italy; marny.fedrigo@unipd.it

**Keywords:** nanoparticle-based drug delivery, myocardial injury, heart failure, cardiomyopathy, evidence mapping, translational nanomedicine, systematic review

## Abstract

Background: Heart failure remains a leading cause of morbidity and mortality, and current therapies rarely repair established myocardial damage. Nanoparticle-based interventions have been investigated across heterogeneous models of myocardial injury, remodeling, cardiomyopathy, and heart failure, but the distribution and translational maturity of this evidence remain unclear. Methods: A systematic search of PubMed, Embase, Scopus, and Web of Science was conducted from database inception to June 2024. Eligible reports were mapped according to disease model, experimental system, carrier-level nanoparticle platform, payload, route, comparator, outcomes, biodistribution, safety assessment, and translational characteristics. Reports of non-therapeutic nanoparticle exposure were retained in a separate contextual safety/toxicology stratum and were not included in the therapeutic evidence-density map. Risk of bias was evaluated using design-appropriate tools. Results: Of 2640 records screened, 157 independent studies met the criteria: 140 in the main therapeutic/platform evidence map and 17 in a separate contextual safety/toxicology stratum. Within the main corpus, polymeric systems were the largest platform class (*n* = 50), followed by inorganic/mineral (*n* = 35), biological/biomimetic (*n* = 24), lipid-based (*n* = 23), carbon-based (*n* = 5), and hybrid/multicomponent systems (*n* = 3). Evidence was concentrated in acute myocardial injury (*n* = 76), while direct same-agent comparisons, long-term safety assessment, repeated dosing, quantitative biodistribution, and clinically aligned heart-failure models remained limited. Conclusions: The field demonstrates substantial formulation diversity and biological activity, but translation is constrained by fragmented characterization, sparse comparative evidence, and incomplete assessment of biological fate and safety.

## 1. Introduction

Cardiovascular diseases remain a leading cause of morbidity and mortality worldwide, and heart failure continues to impose a substantial clinical and socioeconomic burden [1,2,3,4,5]. Heart failure is a complex clinical syndrome that may result from ischemic myocardial injury, chronic pressure or volume overload, cardiotoxicity, metabolic disease, or primary and secondary cardiomyopathies [4,5]. Although myocardial infarction, ischemia–reperfusion injury, cardiac remodeling, and established heart failure are distinct entities, they are frequently connected along a pathophysiological continuum in which cardiomyocyte loss, inflammation, oxidative stress, microvascular dysfunction, fibrosis, and ventricular remodeling contribute to progressive impairment of cardiac function.

Contemporary pharmacological and device-based therapies have markedly improved outcomes in heart failure and selected cardiomyopathies [6,7,8]. Nevertheless, important therapeutic limitations remain, including incomplete delivery of active compounds to injured myocardium, systemic adverse effects, rapid degradation or clearance of biologically active molecules, and limited access to intracellular or subcellular targets. These limitations are particularly relevant during acute myocardial injury and subsequent remodeling, when the therapeutic window may be narrow, and the spatial distribution of pathological processes is heterogeneous.

Nanoparticle-based systems offer a potential means of modifying the pharmacokinetic and biodistribution profiles of therapeutic agents. Their composition, size, surface charge, morphology, degradability, and functionalization can be engineered to encapsulate small molecules, nucleic acids, peptides, proteins, or imaging agents and to provide controlled or stimulus-responsive release [9,10,11,12,13,14,15]. Depending on the platform, nanoparticles may improve payload stability, prolong circulation, facilitate cellular uptake, or enhance retention within injured cardiac tissue. Some nanomaterials also possess intrinsic antioxidant, catalytic, or immunomodulatory activity. These properties, however, do not establish a therapeutic advantage by themselves: benefit must be demonstrated against an appropriate disease control, standard treatment, or the same therapeutic agent administered in a conventional formulation.

Cardiovascular nanomedicine encompasses heterogeneous platforms, including polymeric, lipid-based, inorganic, metallic, silica-based, polysaccharide, protein, biomimetic, and hybrid systems, whose biological performance is highly dependent on their physicochemical characteristics. The same formulation may behave differently across disease models, routes of administration, doses, and stages of myocardial injury. Conversely, apparently similar functional effects may result from different mechanisms, including suppression of inflammation and oxidative stress, inhibition of cell death, modulation of fibrosis, mitochondrial protection, angiogenesis, or improved delivery of a conventional drug. A platform-based comparison is therefore needed to distinguish general *proof-of-concept* effects from reproducible and potentially translatable advantages.

The present systematic review therefore aimed to characterize how nanoparticle platforms have been evaluated across myocardial injury, cardiac remodeling, cardiomyopathy, and heart-failure models; map the relationships among carrier material, payload, disease context, comparator, and outcome; assess the extent of direct evidence against conventional formulations; and identify gaps in biodistribution, safety, manufacturing, and clinical translation.

## 2. Materials and Methods

### 2.1. Protocol, Reporting and Search Strategy

This systematic review was conducted in accordance with the PRISMA 2020 statement [16] and was prospectively registered in PROSPERO (CRD420250587890). PubMed/MEDLINE, Embase, Scopus, and Web of Science were searched from database inception to June 2024 without language restrictions. The study-selection process is summarized in Supplementary Figure S1. Search terms combined concepts related to nanoparticles and nano-enabled delivery systems with terms related to myocardial injury, cardiomyopathy, cardiac dysfunction, and heart failure. The complete database-specific search strategies are provided in Supplementary Table S1.

### 2.2. Eligibility Criteria and Study Selection

Original experimental studies evaluating nanoparticle-based therapeutic interventions in animal, cell-based, or human-derived models of myocardial injury, cardiac remodeling, cardiomyopathy, or heart failure were eligible. Nanoparticles used as therapeutic carriers and nanomaterials with an intended intrinsic therapeutic effect were included.

The eligibility criteria were defined according to a structured PICOS framework and further refined through operational boundaries to ensure consistent classification of disease models, nanoparticle interventions, comparators, outcomes, and contextual safety evidence. These predefined criteria also distinguished therapeutic evidence from studies primarily addressing nanoparticle-related cardiac toxicity (Table S2).

Reviews, editorials, conference abstracts without sufficient data, duplicate or retracted reports, studies without a relevant nanoparticle intervention, and studies without relevant cardiac outcomes were excluded. Titles, abstracts, and full texts were screened according to predefined eligibility criteria, and reasons for full-text exclusion were recorded.

### 2.3. Data Extraction and Classification

Data were extracted using a predefined framework that included experimental model, disease context, nanoparticle platform, payload, physicochemical characteristics, comparator, dose, administration route, follow-up, efficacy outcomes, safety, and biodistribution.

Nanoparticle platforms were classified according to their structural carrier or core as polymeric, lipid-based, inorganic/mineral, biological/biomimetic, carbon-based, or hybrid/multicomponent systems. Disease models and outcomes were grouped into clinically and biologically relevant domains.

### 2.4. Risk-of-Bias Assessment

Risk of bias and methodological reliability were evaluated using design-appropriate assessment tools. Studies performed in laboratory animals were assessed using SYRCLE’s risk-of-bias tool [17], whereas studies conducted exclusively in cell-based or acellular in vitro systems were assessed for methodological reliability using the in vitro ToxRTool [18]. The cross-sectional observational human study was assessed using the Newcastle-Ottawa Scale adapted for cross-sectional studies [19].

For studies combining in vivo and in vitro experiments, a single assessment tool was assigned according to the pivotal study design. Because the primary therapeutic efficacy claims and outcomes in these studies were derived from the in vivo experiments, these studies were assessed using SYRCLE and categorized as in vivo studies for the final risk-of-bias analysis; their accompanying in vitro components were not scored separately. Each study therefore contributed to only one design-specific primary assessment, thereby avoiding double counting.

Reporting completeness of studies assigned to the in vivo category was additionally evaluated using the ARRIVE 2.0 Essential 10 criteria. This assessment was considered complementary to, and distinct from, the SYRCLE risk-of-bias assessment. Full details of tool assignment and categorization of mixed-design studies are provided in the Supplementary Section S3 and Supplementary Table S3.

### 2.5. Evidence-Mapping Strategy

The evidence was synthesized descriptively and organized according to nanoparticle platform, disease model, therapeutic payload, comparator, efficacy outcomes, safety, and translational characteristics. Particular attention was given to direct comparisons between nanoparticle formulations and the corresponding conventional or free therapeutic agents. For the platform-by-assessment-domain evidence-density map, a secondary analytical subset of the main therapeutic/platform corpus was used. Studies were retained when the predefined organ- and outcome-level domains represented in the map could be meaningfully assessed and reliably classified from the extracted data. Studies not permitting a meaningful organ-level assessment were excluded from this specific analysis. This restriction resulted in a subset of 122 studies and did not affect eligibility for or inclusion in the overall systematic review.

Evidence-density maps were used to describe the distribution of the literature and were not interpreted as comparative efficacy rankings. Additional extraction, harmonization, dose-category, and platform classification details are provided in the Supplementary File.

Quantitative meta-analysis was not included in the present manuscript and will be reported separately using a more restricted, outcome-specific analytical framework.

## 3. Results

### 3.1. Study Selection and Overall Characteristics

The database searches identified 3626 records. After removal of 986 duplicates, 2640 records underwent title and abstract screening, of which 2354 were excluded. Full texts were assessed for 286 reports; 128 did not meet the initial eligibility criteria. Manual adjudication therefore considered 158 unique reports. One retracted article was subsequently excluded (Figure S1). Potential multiple reports of the same underlying study were assessed during full-text review. No multiple reports relating to the same study were identified. Of these, 140 formed the main therapeutic/platform evidence corpus, and 17 were retained separately as contextual safety/toxicology evidence (Supplementary Figure S1).

The retained evidence was predominantly preclinical and non-randomized. Of the 157 independent studies, 82 used animal models alone (52.2%), 51 combined animal and cell-based experiments (32.5%), 20 used cell models alone (12.7%), two combined animal experiments with human samples (1.3%), and two involved human material alone (1.3%). Rats and mice were the predominant animal species (Table 1).

Publication year, geographical distribution, experimental system, species, route of administration, dose, and follow-up are summarized in Supplementary Figures S2–S6.

The experimental literature was heterogeneous in intervention design. Intravenous administration was the most frequently reported route (44%), whereas intramyocardial, oral, intraperitoneal, intratracheal, and other routes were less common (Supplementary Figure S5). Exposure ranged from a single administration to several weeks (Supplementary Figures S5 and S6). Considerable heterogeneity was observed in both dose reporting and treatment duration across the included studies. Because *in vitro* concentrations and in vivo doses were expressed using non-comparable units, they were analyzed separately. Most investigations evaluated short- to medium-term exposure, whereas relatively few studies assessed long-term treatment effects (Supplementary Figure S6).

### 3.2. Distribution of Disease Models

**Figure 1:**
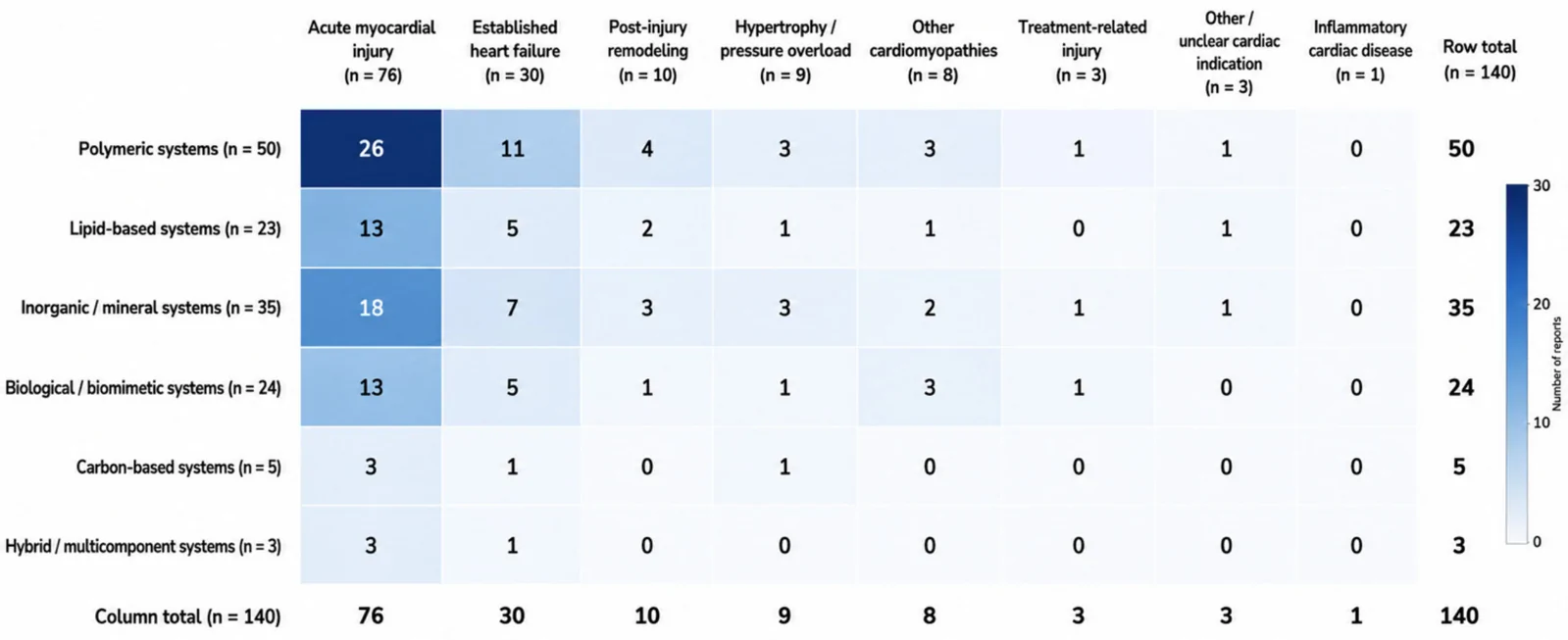
Evidence-density map of nanoparticle platform by cardiovascular disease domain within the 140-study main therapeutic/platform corpus. Cell values represent study counts and must not be interpreted as efficacy rankings. Row totals indicate platform frequencies; column totals indicate disease-domain frequencies.

The 140-report main therapeutic/platform corpus covered a broad but uneven spectrum of cardiovascular disease models. Acute myocardial injury was the dominant domain (*n* = 76; 54.3%), followed by established heart failure (*n* = 30; 21.4%), post-injury remodeling (*n* = 10; 7.1%), hypertrophy or pressure overload (*n* = 9; 6.4%), other cardiomyopathies (*n* = 8; 5.7%), treatment-related injury (*n* = 3; 2.1%), other or unclear cardiac indications (*n* = 3; 2.1%), and inflammatory disease (*n* = 1; 0.7%). This distribution demonstrates that the evidence base is weighted toward acute injury rather than advanced chronic heart failure or primary cardiomyopathy (Figure 1).

### 3.3. Nanoparticle Platform Taxonomy and Evidence Distribution

The original platform labels mixed carrier materials, architectures, payloads, and material subtypes. The revised taxonomy therefore classifies each formulation according to its structural carrier. Dendrimers are treated as polymeric architectures; silver nanoparticles as inorganic/metallic systems; and curcumin as a payload. Cell membrane coating is recorded separately unless the biological material constitutes the principal carrier. Hybrid classification is reserved for structurally integral multicomponent systems.

**Figure 2:**
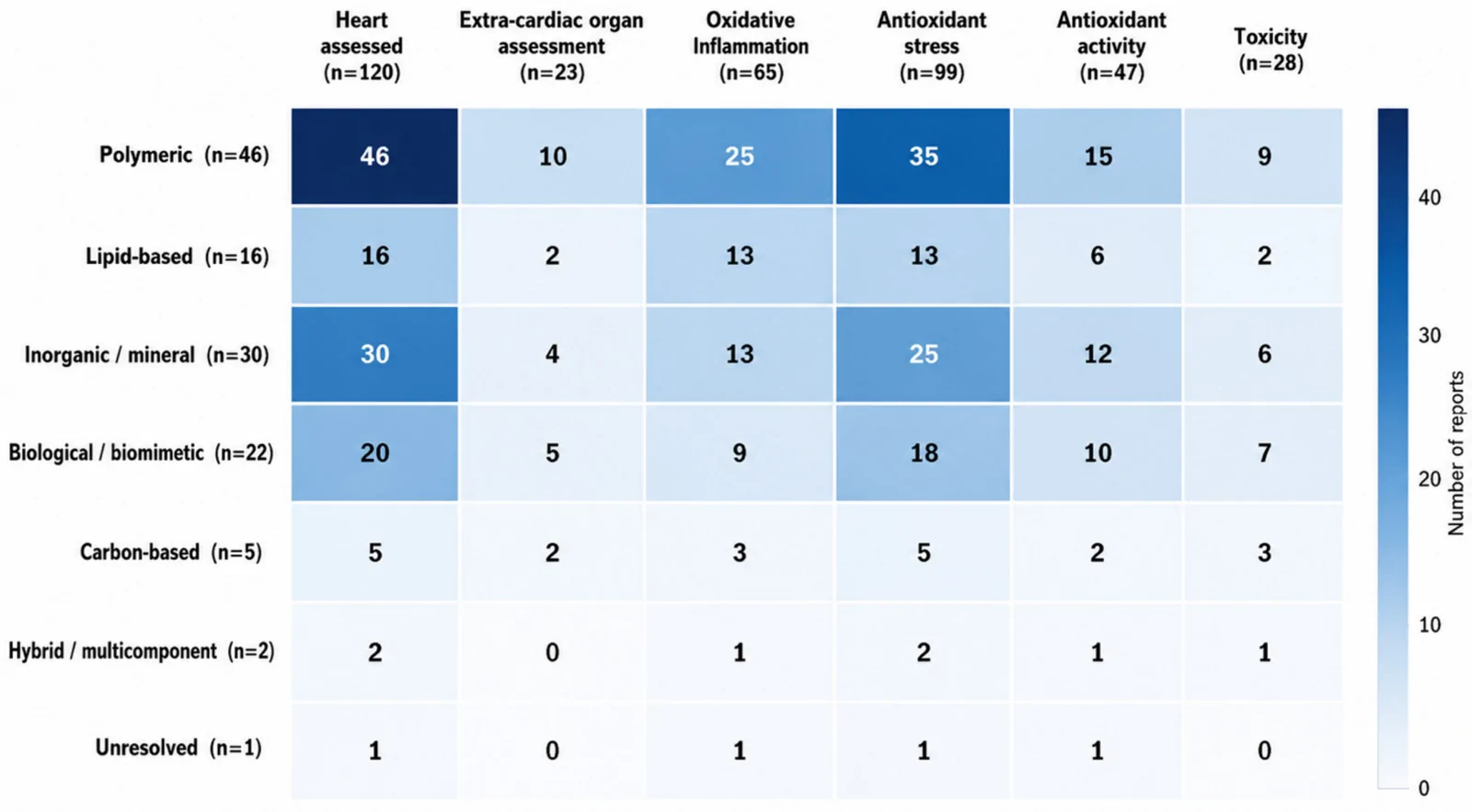
Platform-by-assessment-domain evidence-density map. The map represents the 122-study subset for which organ- and outcome-level assessment was applicable and could be reliably classified from the extracted data. Heart assessed refers to evaluation of cardiac tissue, morphology/pathology, or cardiac functional parameters. Extra-cardiac organ assessment indicates evaluation of at least one non-cardiac organ (liver, kidney, spleen, or lung). The terms Oxidative Inflammation and Antioxidant stress, as displayed in the figure, correspond to the assessment domains recorded in the extraction dataset as inflammation and oxidative stress, respectively. Antioxidant activity refers to assessment of antioxidant-related outcomes, whereas Toxicity refers to active evaluation of adverse or toxic effects. Domains were coded independently and were not mutually exclusive; therefore, individual studies could contribute to more than one column. Cell values indicate evidence density and should not be interpreted as effect magnitude or comparative efficacy.

Within the 140-report main evidence corpus, polymeric systems were most frequent (*n* = 50; 35.7%), followed by inorganic/mineral (*n* = 35; 25.0%), biological/biomimetic (*n* = 24; 17.1%), lipid-based (*n* = 23; 16.4%), carbon-based (*n* = 5; 3.6%), and hybrid/multicomponent platforms (*n* = 3; 2.1%). The 17 contextual safety/toxicology reports comprised 15 inorganic/mineral, one carbon-based, and one hybrid/multicomponent report. Platform for disease and platform for outcome distributions are presented as evidence-density maps in Figure 1 and Figure 2, respectively, and should not be interpreted as comparative efficacy rankings.

### 3.4. Comparative Evidence by Platform

After reclassification by structural carrier, polymeric systems were the largest platform group (*n* = 50), followed by inorganic/mineral (*n* = 35), biological/biomimetic (*n* = 24), lipid-based (*n* = 23), carbon-based (*n* = 5), and hybrid/multicomponent systems (*n* = 3). The main design rationales, observed evidence patterns, and key translational limitations of each nanoparticle platform are summarized in Table 2.

Representative studies are listed in Supplementary Tables S4 and S5. Across platforms, reported effects most commonly involved improved cardiac function or reduced myocardial injury, inflammation, oxidative stress, apoptosis, and fibrosis, together with enhanced angiogenic or regenerative responses.

To further characterize the distribution of organ-level, inflammatory/oxidative, antioxidant, and safety assessments across nanoparticle platforms, a platform-by-assessment-domain evidence-density map was generated (Figure 2). This secondary analysis was restricted to studies for which the predefined organ- and outcome-level domains represented in the map were applicable and could be reliably classified from the extracted data; studies do not allow a meaningful organ-level assessment were excluded from this specific analysis. The resulting subset comprised 122 studies. Assessment domains were coded independently and were not mutually exclusive; therefore, a single study could contribute to more than one column. Accordingly, Figure 2 represents the density and distribution of reported assessments rather than comparative therapeutic efficacy.

Evidence was concentrated in polymeric and inorganic/mineral systems and in acute myocardial injury models. Established heart failure, cardiomyopathies, and carbon-based or hybrid platforms were comparatively less represented.

Polymeric systems included PLGA-, PEG-PLA-, PLGA-PEG-, NIPAAm-MMA-, and dendrimer-based formulations carrying small molecules, nucleic acids, peptides, or growth factors. Reported outcomes included improved ventricular function, smaller infarct or fibrotic areas, reduced inflammatory injury, and enhanced angiogenic or regenerative responses [20,21,22,23,24,25,26,27,28,29,30,31,32,33,34,35,36,37,38,39,40,41,42,43,44,45,46,47,48,49,50,51,52,53,54,55,56,57,58,59,60,61,62,63,64,65,66,67,68].

Lipid-based systems included liposomes, solid lipid nanoparticles, nanostructured lipid carriers, and related formulations used for small-molecule and nucleic-acid delivery. Studies reported improved drug exposure and favorable effects on cardiac function, inflammation, fibrosis, and cardiotoxic injury [69,70,71,72,73,74,75,76,77,78,79,80,81,82,83,84,85,86,87,88,89,90,91].

Inorganic/mineral systems comprised gold, silver, iron oxide, cerium oxide, silica, calcium-based, selenium-containing, and catalytic nanomaterials. Reported applications included antioxidant and anti-inflammatory activity, infarct and remodeling reduction, magnetic guidance, targeted delivery, and imaging [92,93,94,95,96,97,98,99,100,101,102,103,104,105,106,107,108,109,110,111,112,113,114,115,116,117,118,119,120,121,122,123,124].

Biological and biomimetic platforms included polysaccharide- or protein-based carriers, cell-derived systems, and membrane-coated nanoparticles. Reported applications involved immune-cell targeting, modulation of inflammation and fibrosis, angiogenesis, and delivery to injured myocardium [125,126,127,128,129,130,131,132,133,134,135,136,137,138,139,140,141,142,143,144,145,146,147].

Carbon-based and hybrid/multicomponent systems were represented by small and heterogeneous datasets. These formulations combined carrier, imaging, catalytic, magnetic, or stimuli-responsive functions, but the limited number of studies precluded class-level conclusions [148,149,150,151,152,153,154,155].

Overall, the literature showed recurrent preclinical signals of cardiac protection, but the evidence did not support an efficacy hierarchy among material classes because disease models, payloads, routes, doses, follow-up, and outcome selection differed substantially across studies.

### 3.5. Direct Comparisons with Conventional Therapy

Most included studies compared nanoparticle-based interventions with untreated or vehicle-treated disease controls, whereas direct comparisons with the same active agent administered in a conventional or free formulation were uncommon. Consequently, although the available studies frequently demonstrated therapeutic activity, evidence supporting a formulation-specific advantage attributable to nanoparticle delivery remained limited. Direct comparative evidence was particularly sparse for claims of improved bioavailability, enhanced cardiac targeting, dose sparing, prolonged action, reduced systemic toxicity, and superior therapeutic efficacy.

### 3.6. Safety, Biodistribution, and Off-Target Effects

Safety and toxicity were less consistently evaluated than efficacy across the included evidence base. Seventeen non-therapeutic exposure or toxicology reports were retained as a separate contextual safety/toxicology stratum and were not included in the 140-study therapeutic/platform evidence map (Figure 1). Within therapeutic studies, safety-related assessments included cell viability, oxidative stress, serum chemistry, organ histology, inflammation, and hemodynamic parameters, although the extent and type of safety assessment varied considerably across studies.

Within the contextual safety/toxicology stratum, 15 of the 17 studies involved inorganic/mineral nanoparticles, while one investigated a carbon-based system and one a hybrid/multicomponent system. Reported adverse effects varied according to nanoparticle composition, particle size, dose, surface properties, administration route, and exposure duration. Reported cardiac and systemic effects included oxidative stress, inflammation, lysosomal or mitochondrial dysfunction, vascular impairment, apoptosis, cardiac remodeling, and hypertrophic responses [156,157,158,159,160,161,162,163,164,165,166,167,168,169,170] (Supplementary Table S4).

Studies evaluating nanoparticle-based interventions for the prevention or treatment of doxorubicin- or chemotherapy-associated cardiac injury were classified within the therapeutic evidence corpus rather than the contextual safety/toxicology stratum. In these studies, cardio protection represented the therapeutic endpoint and was considered separately from assessment of nanoparticle carrier safety.

Biodistribution and off-target organ assessment were heterogeneous across the included studies. The heart represented the intended target in most therapeutic studies, whereas assessment of the liver, spleen, kidney, lung, and other organs was less consistently reported. Quantitative biodistribution methods, assessment time points, and reporting approaches also varied across studies, limiting direct comparison of nanoparticle distribution and persistence (Supplementary Table S3).

Administration routes were heterogeneous, with intravenous administration being the most frequently reported approach, followed by intramyocardial, oral, intraperitoneal, intratracheal, and other routes (Supplementary Figure S5). Dose reporting and exposure duration were similarly heterogeneous, with in vitro concentrations and in vivo doses reported using non-comparable units (Supplementary Figure S6).

Long-term follow-up and repeated-dose studies were uncommon, and physicochemical characterization and active assessment of off-target organ toxicity were inconsistently reported. Evaluation of immune, complement, coagulation, hemolytic, reproductive, and genotoxic effects was limited. Protein-corona formation and its relationship with biodistribution, clearance, and immunological responses were also rarely assessed directly.

### 3.7. Risk of Bias and Evidence Gaps

Risk of bias was assessed for 155 of the 157 studies. Among the 136 in vivo studies assessed using SYRCLE, selective outcome reporting was judged at low risk in all studies (100%), and other bias was judged at low risk in most (69%). In contrast, allocation concealment and random housing were not reported in any study, and caregiver/investigator blinding was reported in only 5%. Sequence generation (46%), baseline comparability (34%), and outcome-assessor blinding (24%) were reported more frequently but remained incomplete. Consequently, the overall risk of bias was unclear for essentially all studies assessed with SYRCLE, largely reflecting insufficient reporting of methodological safeguards rather than evidence of demonstrable methodological flaws. Reporting completeness (ARRIVE 2.0) discriminated more finely (median 6/10; IQR 5–7; range 3–9): experimental procedures (100%), statistical methods (99%), and study design (88%) were well reported, whereas randomization (40%), blinding (29%), sample-size justification (10%), and inclusion/exclusion criteria (4%) were not. All 17 *in vitro* studies were classified as reliable (ToxRTool Klimisch category 1). The cross-sectional observational human study was rated as having moderate-to-good methodological quality using the Newcastle–Ottawa Scale (7/10). Details of the design-specific assessment tools and study categorization are provided in the Supplementary Section S3 and Supplementary Table S3.

## 4. Discussion

### 4.1. Strengths and Principal Findings

The principal strength of this review is its structured separation of disease context, nanoparticle carrier, therapeutic payload, comparator strength, and translational evidence across 157 independent studies. By distinguishing the 140-study therapeutic/platform corpus from 17 contextual safety/toxicology studies and organizing formulations according to their structural carrier, the review provides an evidence map rather than a simple catalogue of positive findings. This approach identifies where the field is concentrated, where formulation-specific benefit has actually been tested, and which translational domains remain insufficiently evaluated.

The evidence base was dominated by acute myocardial injury and by polymeric, inorganic/mineral, lipid-based, and biological/biomimetic platforms. Across these categories, studies frequently reported improved cardiac function, reduced myocardial injury, and modulation of inflammation, oxidative stress, cell death, fibrosis, and angiogenesis. However, the literature remained predominantly preclinical and heterogeneous, and no material class could be considered superior on the basis of the available evidence.

### 4.2. Disease Context and Platform Interpretation

Myocardial infarction, ischemia-reperfusion injury, post-injury remodeling, established heart failure, and cardiomyopathies are biologically related but clinically distinct. The predominance of acute injury models means that the strongest evidence concerns cardio protection and early remodeling rather than reversal of advanced chronic heart failure. Therapeutic timing should therefore be interpreted explicitly, because interventions administered before or immediately after ischemia address a different clinical question from treatments initiated after ventricular dysfunction is established.

Platform labels also encompass substantial internal diversity. Formulations within the same broad class may differ in size, surface chemistry, payload, targeting ligand, release kinetics, route, degradation, and persistence. The biologically relevant unit is therefore the complete formulation, not the material category alone; carrier, payload, coating, and functional role must be reported and interpreted separately.

### 4.3. Formulation-Specific Advantage and Biological Mechanisms

The most important comparative limitation was the scarcity of matched head-to-head studies. Comparisons with untreated or vehicle-treated disease demonstrate biological activity but do not establish that nanoparticle delivery is superior to the same agent in free or conventional form. Claims of improved bioavailability, cardiac targeting, dose sparing, prolonged action, reduced toxicity, or superior efficacy require dose- and route-matched comparators, quantitative pharmacokinetics or biodistribution, and direct statistical comparison.

To facilitate the interpretation of formulation-specific claims, Table 3 summarizes the minimum evidentiary requirements for demonstrating an advantage of nanoparticle-based delivery over conventional therapy.

Reported mechanisms converged on inflammation, oxidative stress, mitochondrial dysfunction, apoptosis, autophagy, pyroptosis, ferroptosis, fibrosis, and angiogenesis [20,69,92,125,148,153].

This interpretation is consistent with the broader nanomedicine literature; Wan et al. [171] emphasized that nanoparticle strategies may influence interconnected cell-death programs rather than a single isolated pathway. A recent non-cardiac proof-of-concept study by Qian et al. [172], using manganese-substituted Prussian blue nanocubes, further illustrated how autophagy activation, reactive-oxygen-species scavenging, and suppression of inflammation can converge to limit fibrotic remodeling. Although derived from liver fibrosis, this work supports the biological plausibility of multi-pathway nano-interventions while not constituting direct cardiovascular evidence. These pathways are biologically plausible, but mechanistic association should not be equated with causal proof. Observed effects may derive from the payload, intrinsic nanomaterial activity, altered biodistribution, or their combination.

### 4.4. Safety and Translational Readiness

Safety evidence was less mature than efficacy evidence. Short-term cell viability, serum chemistry, or limited organ histology cannot establish long-term biocompatibility, and absence of reported harm should be considered non-assessment unless safety outcomes were actively measured. The contextual toxicology stratum showed that adverse effects were material- and exposure-specific, particularly for persistent inorganic/mineral systems.

Translation also depends on biological identity and manufacturing control. Protein-corona formation may alter targeting, immune recognition, biodistribution, and clearance [173,174], while incomplete assessment of repeated dosing, organ accumulation, degradation, and excretion limits confidence in long-term safety. In this context, Li et al. [175] reviewed interfacial self-assembly approaches that can generate tunable ordered nanostructures through non-covalent and interface-driven interactions. These strategies also underscore the need to control assembly conditions, surface chemistry, structural uniformity, and production reproducibility. Reproducible control of particle size, surface composition, payload content, release, aggregation, sterility, residual components, and storage stability will be essential for clinical development.

### 4.5. Priorities for Future Research

Future studies should prioritize clinically aligned disease stages, matched same-agent comparators, formulation-specific physicochemical characterization, quantitative pharmacokinetics and biodistribution, repeated-dose designs, and active long-term safety assessment. Standardized reporting should allow independent reconstruction of carrier composition, payload exposure, targeting performance, and manufacturing reproducibility.

## 5. Limitations

The review encompasses heterogeneous diseases, experimental systems, intervention purposes, and nanoparticle architectures. Manual adjudication yielded a stable corpus of 157 independent studies, each represented by one report; none of the 59 candidate linkage pairs arose from the same underlying study. Seventeen exposure/toxicology studies were deliberately retained as contextual safety evidence but excluded from the 140-study therapeutic evidence map; this improves conceptual clarity but does not replace a formal toxicological review. Several studies provide insufficient information to distinguish carrier, payload, and coating. Direct comparisons, quantitative biodistribution, and active long-term safety assessment remain sparse, limiting formulation-specific and translational conclusions.

## 6. Conclusions

Nanoparticle-based cardiac research is broad but unevenly distributed and remains translationally immature. The evidence is concentrated in acute ischemic models and demonstrates extensive formulation innovation, yet few studies establish a reproducible advantage over conventional delivery or provide adequate long-term fate and safety data. A carrier-level taxonomy, matched comparative designs, and standardized translational reporting are necessary to identify which formulations warrant further development.

## Figures and Tables

**Table 1:** Characteristics and distribution of the 157 included independent studies.

Domain	Category	Studies, *n*	Percentage
**Evidence stratum**	Main therapeutic/platform evidence	140	89.2%
**Evidence stratum**	Contextual safety/toxicology evidence	17	10.8%
**Model type**	Animal only	82	52.2%
**Model type**	Animal + cell-based	51	32.5%
**Model type**	Cell-based only	20	12.7%
**Model type**	Animal + human material	2	1.3%
**Model type**	Human material only	2	1.3%
**Disease domain (main corpus)**	Acute myocardial injury	76	54.3%
**Disease domain (main corpus)**	Established heart failure	30	21.4%
**Disease domain (main corpus)**	Post-injury remodeling	10	7.1%
**Disease domain (main corpus)**	Hypertrophy/pressure overload	9	6.4%
**Disease domain (main corpus)**	Other cardiomyopathies	8	5.7%
**Disease domain (main corpus)**	Treatment-related injury	3	2.1%
**Disease domain (main corpus)**	Other/unclear cardiac indication	3	2.1%
**Disease domain (main corpus)**	Inflammatory cardiac disease	1	0.7%
**Platform (main corpus)**	Polymeric	50	35.7%
**Platform (main corpus)**	Inorganic/mineral	35	25.0%
**Platform (main corpus)**	Biological/biomimetic	24	17.1%
**Platform (main corpus)**	Lipid-based	23	16.4%
**Platform (main corpus)**	Carbon-based	5	3.6%
**Platform (main corpus)**	Hybrid/multicomponent	3	2.1%

Percentages for evidence strata and model types use 157 as the denominator; disease-domain and platform percentages use the 140-study main therapeutic/platform corpus.

**Table 2:** Comparative synthesis of nanoparticle platforms represented in the 140-study main evidence corpus.

Platform (*n*, Main Corpus)	Principal Design Rationale and Applications	Observed Evidence Pattern	Key Limitations/Translational Issues
**Polymeric (*n* = 50)**	Controlled release; protection of labile small molecules, nucleic acids, peptides, and growth factors; surface functionalization.	Largest and broadest therapeutic cluster; frequent signals in acute injury and remodeling models.	Marked formulation heterogeneity; loading/release reproducibility; degradation products; manufacturing scale-up.
**Inorganic/mineral (*n* = 35)**	Intrinsic catalytic, magnetic, imaging, antioxidant, or delivery functions.	Signals for oxidative-injury modulation, targeting, imaging, and magnetic cell guidance.	Composition-specific persistence and toxicity; organ accumulation; dissolution/excretion; long-term fate.
**Biological/biomimetic (*n* = 24)**	Extracellular vesicles, cell-derived carriers, membrane-coated systems, and endogenous-like interactions.	Potential for cell communication, immune-cell targeting, and regenerative delivery.	Source variability; cargo attribution; immunogenicity; purification, potency, and batch consistency.
**Lipid-based (*n* = 23)**	Delivery of hydrophilic/hydrophobic drugs and nucleic acids; altered exposure.	Cardioprotective, anti-inflammatory, and antifibrotic signals across mainly preclinical models.	Oxidation, leakage, storage stability, complement activation, and hepatic/splenic uptake.
**Carbon-based (*n* = 5)**	High surface area, functionalization, conductive, or carrier properties.	Sparse, heterogeneous evidence; no class-level efficacy inference.	Bio persistence, oxidative injury, morphology-specific toxicity, and limited long-term evidence.
**Hybrid/multicomponent (*n* = 3)**	Integration of structural, targeting, imaging, or stimuli-responsive functions.	Proof-of-concept multifunctionality in very small datasets.	Complex characterization, stability, batch consistency, regulatory definition, and attribution of activity/toxicity.

Counts describe evidence density, not comparative efficacy. Platform categories refer to the structural carrier/core rather than the payload.

**Table 3:** Interpretive framework for establishing formulation-specific advantage over conventional therapy.

Claimed Advantage	Minimum Evidence Required	Comparator/Design Requirement	Interpretive Rule
**Improved bioavailability**	Pharmacokinetic exposure, concentration-time profile, and relevant tissue exposure.	Same active agent; matched dose, route, schedule, and sampling.	Efficacy versus untreated disease alone does not demonstrate improved bioavailability.
**Enhanced cardiac targeting**	Quantitative, time-resolved cardiac and off-target biodistribution.	Free-agent or non-targeted formulation comparator with matched exposure.	Cellular uptake or a single terminal image is insufficient to establish targeting advantage.
**Dose sparing**	Comparable or superior efficacy at a lower active-agent exposure.	Dose-ranging, same-agent comparison with equivalent outcome timing.	Lower nominal nanoparticle mass is not dose-sparing unless active payload exposure is defined.
**Prolonged action**	Release/exposure kinetics and durable functional effect across prespecified time points.	Matched active agent and follow-up; preferably repeated measurements.	A later endpoint without time-course evidence cannot establish prolonged action.
**Reduced systemic toxicity**	Active safety assessment at matched dose or systemic exposure.	Same-agent comparator; organ, immune, hematologic, and clinical-chemistry assessment as appropriate.	Absence of reported harm is classified as non-assessment, not evidence of safety.
**Superior therapeutic efficacy**	Direct statistical comparison between nano-formulation and conventional delivery.	Randomized or otherwise balanced same-agent groups with matched dose, route, timing, and follow-up.	Separate significance versus disease control is not evidence of a nano-versus-conventional difference.

This table defines the evidentiary standard for the planned comparator audit; it does not imply that all included studies met these requirements.

## Data Availability

No new data were created or analyzed in this study. Data sharing is not applicable to this article.

## Supplementary Materials

The following supporting information can be downloaded at: https://www.mdpi.com/article/10.3390/biom16091245/s1, Figure S1: PRISMA 2020 flow diagram; Table S1. Complete database-specific search strategies.; Table S2. Eligibility criteria and operational boundaries for the systematic review; Table S3. Design-specific assignment of risk-of-bias and methodological-quality assessment tools; Table S4. Representative studies of nanoparticle-based therapies across cardiovascular indications, with key endpoints and mechanistic features; Table S5. Toxicity and safety-related nanoparticle studies in cardiovascular preclinical research; Figures S2–S6. Descriptive characteristics of the retained evidence base [17,18,84,176,177].

## Abbreviations

The following abbreviations are used in this manuscript:

ACE	Angiotensin-converting enzyme
AMI	Acute myocardial infarction
AR	Adrenergic receptor
AuNPs	Gold nanoparticles
CAT	Catalase
CaMKII	Calcium/calmodulin-dependent protein kinase II
ColCaNPs	Colchicine-containing calcium carbonate nanoparticles
CaPs	Calcium phosphate nanoparticles
CC	Cardiac output
CD	Cluster of differentiation
CHF	Chronic heart failure
CIs	Confidence intervals
CMECs	Cardiac microvascular endothelial cells
CSF-1	Colony-stimulating factor 1
CSWT	Cardiac shock wave therapy
CVDs	Cardiovascular diseases
CUR-NPs	Curcumin-loaded nanoparticles
dp/dt max	Maximum rate of rise of left ventricular pressure
EF	Ejection fraction
ERK1/2	Extracellular signal-regulated kinase 1/2
FGF1	Fibroblast growth factor 1
GPx	Glutathione peroxidase
GSH	Reduced glutathione
HA	Hyaluronic acid
HF	Heart failure
HFpEF	Heart failure with preserved ejection fraction
ICD	Implantable cardioverter-defibrillator
IL	Interleukin
IONPs	Iron oxide nanoparticles
IQR	Interquartile range
JNK	c-Jun N-terminal kinase
KLF2	Krüppel-like factor 2
LDL	Low-density lipoprotein
LDE-MTX	Lipid core nanoparticles carrying methotrexate
LNPs	Lipid nanoparticles
LV	Left ventricle
LVEDP	Left ventricular end-diastolic pressure
LVEDV	Left ventricular end-diastolic volume
LVEF	Left ventricular ejection fraction
LVESV	Left ventricular end-systolic volume
MAPK	Mitogen-activated protein kinase
MDA	Malondialdehyde
MI	Myocardial infarction
MSCs	Mesenchymal stem cells
MSNs	Mesoporous silica nanoparticles
mPTP	Mitochondrial permeability transition pore
NC	Nanocerium (cerium oxide nanoparticles)
NF-κB	Nuclear factor kappa-light-chain-enhancer of activated B cells
NIL10	IL-10 receptor-binding nanoparticle formulation
NIPAAm-MMA NPs	N-isopropylacrylamide-methyl methacrylate nanoparticles
NLCs	Nanostructured lipid carriers
NO	Nitric oxide
NPs	Nanoparticles
NPLD	Non-pegylated liposomal doxorubicin
PAMPs	Pathogen-associated molecular patterns
PAMAM	Poly(amidoamine) dendrimer nanoparticles
PICOS	Population, Intervention, Comparison, Outcome, Study design
PLGA	Poly(lactic-co-glycolic acid)
Pt-N-C	Platinum–nitrogen–carbon nanozyme
PRISMA	Preferred Reporting Items for Systematic Reviews and Meta-Analyses
R	R statistical software
RCTs	Randomized controlled trials
ROBINS-I	Risk Of Bias In Non-randomized Studies of Interventions
ROS	Reactive oxygen species
SMD	Standardized mean difference
SOD	Superoxide dismutase
SeNPs	Selenium nanoparticles
SiNPs	Silica nanoparticles
SLNs	Solid lipid nanoparticles
SPIONs	Superparamagnetic iron oxide nanoparticles
STAT3	Signal transducer and activator of transcription 3
SYRCLE	Systematic Review Centre for Laboratory Animal Experimentation
TGF-β	Transforming growth factor beta
TGF-β3	Transforming growth factor beta 3
TLR4	Toll-like receptor 4
TNF-α	Tumor necrosis factor alpha
VAD	Ventricular assist device
VEGF	Vascular endothelial growth factor
