# Supplementary material for: Nanoparticle-Based Therapies for Myocardial Injury and Heart Failure: A Systematic Review and Translational Appraisal of Preclinical Evidence
Source: Biomolecules. 2026 Aug 27;16(9):1245. doi: 10.3390/biom16091245 (PMC13604711; doi:10.3390/biom16091245)
Supplement: Supplementary file 1 [file biomolecules-16-01245-s001.zip › Supplementary_Materials_REVISED_Biomolecules_4415704_clean copy.pdf]

SUPPLEMENTARY MATERIALS

Nanoparticle-Based Therapies for Myocardial Injury and Heart Failure

A Systematic Review and Translational Appraisal of Preclinical Evidence

Ayesha Jabeen, Ilaria Barison, Honoria Ocagli, Bruna Fata, Diego Perazzolo, Cristina Basso, Roberto Luisetto, Silvia Pozzo, Fabrizio Mancin, Enrico Grisan, Dario Gregori, Annalisa Angelini, Marny Fedrigo and Chiara Castellani

Organization of the Supplementary Materials

Supplementary Figure S1. PRISMA 2020 flow diagram

Supplementary Table S1. Complete database-specific search strategies.

Supplementary Table S2. Eligibility criteria and operational boundaries for the systematic review

Supplementary Table S3. Design-specific assignment of risk-of-bias and methodological-quality assessment tools

Supplementary Table S4. Representative studies of nanoparticle-based therapies across cardiovascular indications, with key endpoints and mechanistic features

Supplementary Table S5. Toxicity and safety-related nanoparticle studies in cardiovascular preclinical research

Supplementary Figures S2-S6. Descriptive characteristics of the retained evidence base.

Supplementary Methods

S1. Search Strategy

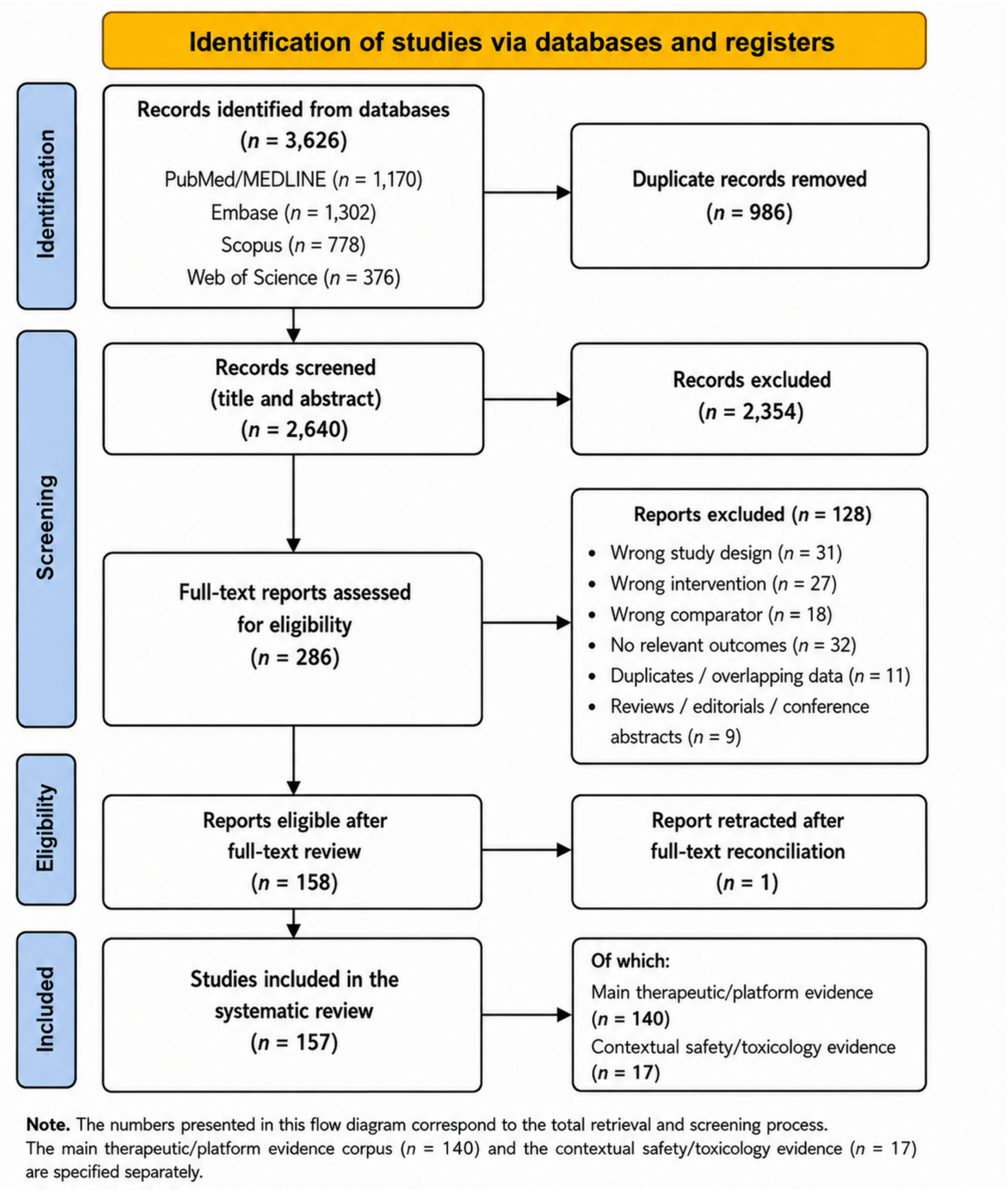

Supplementary Figure S1. *PRISMA 2020 flow diagram of study identification, screening, eligibility assessment, and inclusion.*

PubMed/MEDLINE, Embase, Scopus, and Web of Science were searched from database inception to June 2024 without language restrictions. Search concepts combined controlled vocabulary and free-text terms for nanoparticles and nano-enabled delivery systems with terms related to myocardial injury, cardiomyopathy, cardiac dysfunction, and heart failure. The complete database-specific strategies are provided in Supplementary Table S1.

Supplementary Table S1. Database search strategy

Database	Complete search strategy
PubMed	("Nanoparticles"[MeSH Terms] OR "Nanoparticles"[Title/Abstract] OR "Nanoparticle"[Title/Abstract] OR "Nanocrystalline Materials"[Title/Abstract] OR "Nanocrystalline Material"[Title/Abstract] OR "Nanocrystals"[Title/Abstract] OR "Nanocrystal"[Title/Abstract] OR "Nanoparticle Drug Delivery System"[MeSH Terms] OR "Nano Drug Delivery Systems"[Title/Abstract] OR "nano drug delivery system"[Title/Abstract] OR "nano drug delivery system"[Title/Abstract] OR "Nano Delivery System"[Title/Abstract] OR "Nano Delivery Systems"[Title/Abstract] OR "NDDSs"[Title/Abstract] OR "Nanoparticle Based Drug Delivery System"[Title/Abstract]) AND ("Cardiomyopathies"[MeSH Terms] OR "Myocardial Disease"[Title/Abstract] OR "Myocardial Diseases"[Title/Abstract] OR "Myocardiopathies"[Title/Abstract] OR "Myocardiopathy"[Title/Abstract] OR "Primary Cardiomyopathies"[Title/Abstract] OR "Primary Cardiomyopathy"[Title/Abstract] OR "Primary Myocardial Disease"[Title/Abstract] OR "Primary Myocardial Diseases"[Title/Abstract] OR "Secondary Cardiomyopathies"[Title/Abstract] OR "Secondary Cardiomyopathy"[Title/Abstract] OR "Secondary Myocardial Disease"[Title/Abstract] OR "Secondary Myocardial Diseases"[Title/Abstract] OR "heart failure, systolic"[MeSH Terms] OR "heart failure reduced ejection fraction"[Title/Abstract] OR "Systolic Heart Failure"[Title/Abstract] OR "heart failure, diastolic"[MeSH Terms] OR "Diastolic Heart Failures"[Title/Abstract] OR "Heart Failure"[MeSH Terms] OR "Heart Failure"[Title/Abstract] OR "Cardiac Failure"[Title/Abstract] OR "Heart Decompensation"[Title/Abstract] OR "Congestive Heart Failure"[Title/Abstract] OR "heart failure congestive"[Title/Abstract] OR "heart failure right sided"[Title/Abstract] OR "heart failure right sided"[Title/Abstract] OR "heart failure left sided"[Title/Abstract] OR "heart failure left sided"[Title/Abstract] OR "Myocardial Failure"[Title/Abstract])
Scopus	(TITLE-ABS ("cardiomyopathies" OR " cardiomyopathies" OR "cardiomyopathy" OR "myocardial disease" OR "myocardial diseases" OR "myocardiopathies" OR "myocardiopathy" OR "primary cardiomyopathies" OR "primary cardiomyopathy" OR "primary myocardial disease" OR "primary myocardial diseases" OR "secondary cardiomyopathies" OR "secondary cardiomyopathy" OR "secondary myocardial disease" OR "secondary myocardial diseases" OR "heart failure, systolic" OR "heart failures, systolic" OR "heart failure, reduced ejection fraction" OR "systolic heart failure" OR "heart failure, diastolic" OR "diastolic heart failures" OR " heart failure, preserved ejection fraction" OR "heart failure, normal ejection fraction" OR "heart failure" OR "cardiac failure" OR "heart decompensation" OR "congestive heart failure" OR "heart failure, congestive" OR "heart failure, right-sided" OR "heart failure, right sided" OR "heart failure, left-sided" OR "heart failure, left sided" OR "myocardial failure")) AND (TITLE-ABS ("nanoparticles" OR "nanoparticle" OR "nanocrystalline materials" OR "nanocrystalline material" OR "nanocrystals" OR "nanocrystal" OR "nanoparticle drug delivery system" OR "nano drug delivery systems" OR "nano- drug delivery system" OR "nano drug delivery system" OR "nano delivery system" OR "nano delivery systems" OR "nddss" OR "nanoparticle-based drug delivery system"))
Embase	Cardiomyopathies.ab,ti or exp cardiomyopathy/ or exp myocardial disease/ or Cardiomyopathies.ab,ti. or Cardiomyopathy.ab,ti or Myocardial Disease.ab,ti. or Myocardial Diseases.ab,ti. or Myocardiopathies.ab,ti or Myocardiopathy.ab,ti. or Primary Cardiomyopathies.ab,ti. or Primary Cardiomyopathy.ab,ti. or Primary Myocardial Disease.ab,ti. or Primary Myocardial Diseases.ab,ti. or Secondary Cardiomyopathies.ab,ti. or Secondary Cardiomyopathy.ab,ti. or Secondary Myocardial Disease.ab,ti. or Secondary Myocardial Diseases.ab,ti. or exp systolic heart failure/ or Systolic Heart Failure.ab,ti. or exp diastolic heart failure/ or Diastolic Heart Failures.ab,ti. or Heart Failure.mp. or exp heart failure/ or Heart Failure.ab,ti. or Cardiac Failure.ab,ti. or Heart Decompensation.ab,ti. or Congestive Heart Failure.ab,ti. or Myocardial Failure.ab,ti. AND Nanoparticles.ab,ti. or exp nanoparticle/ or Nanoparticles.ab,ti. or Nanoparticle.ab,ti. or Nanocrystalline Materials.ab,ti. or Nanocrystalline Material.ab,ti. or Nanocrystals.ab,ti. or Nanocrystal.ab,ti. or Nanoparticle Drug Delivery System.mp. or exp nanocarrier/ or Nano Drug Delivery Systems.ab,ti. or Nano-Drug Delivery System.ab,ti. or Nano Delivery System.ab,ti. or Nano Delivery Systems.ab,ti. or NDDSs.ab,ti. or Nanoparticle Based Drug Delivery System.ab,ti.
Web of Science	"Cardiomyopathies" OR " Cardiomyopathies" OR "Cardiomyopathy" OR "Myocardial Disease" OR "Myocardial Diseases" OR "Myocardiopathies" OR "Myocardiopathy" OR "Primary Cardiomyopathies" OR "Primary Cardiomyopathy" OR "Primary Myocardial Disease" OR "Primary Myocardial Diseases" OR "Secondary Cardiomyopathies" OR "Secondary Cardiomyopathy" OR "Secondary Myocardial Disease" OR "Secondary Myocardial Diseases" OR "Heart Failure, Systolic" OR "Heart Failures, Systolic" OR "Heart Failure, Reduced Ejection Fraction" OR "Systolic Heart Failure" OR "Heart Failure, Diastolic" OR "Diastolic Heart Failures" OR " Heart Failure, Preserved Ejection Fraction" OR "Heart Failure, Normal Ejection Fraction" OR "Heart Failure" OR "Cardiac Failure" OR "Heart Decompensation" OR "Congestive Heart Failure" OR "Heart Failure, Congestive" OR "Heart Failure, Right-Sided" OR "Heart Failure, Right Sided" OR "Heart Failure, Left-Sided" OR "Heart Failure, Left Sided" OR "Myocardial Failure" (Title) or "Cardiomyopathies" OR " Cardiomyopathies" OR "Cardiomyopathy" OR "Myocardial Disease" OR "Myocardial Diseases" OR "Myocardiopathies" OR "Myocardiopathy" OR "Primary Cardiomyopathies" OR "Primary Cardiomyopathy" OR "Primary Myocardial Disease" OR "Primary Myocardial Diseases" OR "Secondary Cardiomyopathies" OR "Secondary Cardiomyopathy" OR "Secondary Myocardial Disease" OR "Secondary Myocardial Diseases" OR "Heart Failure, Systolic" OR "Heart Failures, Systolic" OR "Heart Failure, Reduced Ejection Fraction" OR "Systolic Heart Failure" OR "Heart Failure, Diastolic" OR "Diastolic Heart Failures" OR " Heart Failure, Preserved Ejection Fraction" OR "Heart Failure, Normal Ejection Fraction" OR "Heart Failure" OR "Cardiac Failure" OR "Heart Decompensation" OR "Congestive Heart Failure" OR "Heart Failure, Congestive" OR "Heart Failure, Right-Sided" OR "Heart Failure, Right Sided" OR "Heart Failure, Left-Sided" OR "Heart Failure, Left Sided" OR "Myocardial Failure" (Abstract) AND "Nanoparticles" OR "Nanoparticle" OR "Nanocrystalline Materials" OR "Nanocrystalline Material" OR "Nanocrystals" OR "Nanocrystal" OR "Nanoparticle Drug Delivery System" OR "Nano Drug Delivery Systems" OR "Nano-Drug Delivery System" OR "Nano Drug Delivery System" OR "Nano Delivery System" OR "Nano Delivery Systems" OR "NDDSs" OR "Nanoparticle Based Drug Delivery System" (Title) or "Nanoparticles" OR "Nanoparticle" OR "Nanocrystalline Materials" OR "Nanocrystalline Material" OR "Nanocrystals" OR "Nanocrystal" OR "Nanoparticle Drug Delivery System" OR "Nano Drug Delivery Systems" OR "Nano-Drug Delivery System" OR "Nano Drug Delivery System" OR "Nano Delivery System" OR "Nano Delivery Systems" OR "NDDSs" OR "Nanoparticle Based Drug Delivery System" (Abstract)

S2. Eligibility Criteria

Original experimental reports evaluating a nanoparticle-based therapeutic intervention in an eligible model of myocardial injury, cardiac remodeling, heart failure, or cardiomyopathic disease were considered for the main evidence corpus. Eligible interventions included nanoparticles used as therapeutic carriers and nanomaterials with an intended intrinsic therapeutic effect. Reports in which nanoparticle exposure caused or modified cardiac injury without a therapeutic intent were retained separately as contextual safety/toxicology evidence and did not contribute to the therapeutic evidence-density map. Comparators comprised untreated disease controls, vehicle- or placebo-treated controls, standard therapy, or the same therapeutic agent administered in a conventional non-nanoparticle formulation, where available. Eligible outcomes included measures of cardiac function, myocardial injury, ventricular remodeling, fibrosis, cardiomyocyte structure, inflammation, oxidative stress, cell death, angiogenesis, safety, biodistribution, and targeting. Reviews, editorials, conference abstracts without sufficient data, duplicate reports, retracted articles, reports without a relevant nanoparticle intervention, and reports without relevant cardiac outcomes were excluded. The eligibility criteria were defined according to a structured PICOS framework and further refined through operational boundaries to ensure consistent classification of disease models, nanoparticle interventions, comparators, outcomes, and contextual safety evidence. These predefined criteria also distinguished therapeutic evidence from studies primarily addressing nanoparticle-related cardiac toxicity (Table S2).

Supplementary Table S2. Eligibility criteria and operational boundaries for the systematic review

PICOS element	Inclusion criteria	Exclusion criteria	Operational clarification
Population / model	In vivo animal models; cell-based systems; eligible human material relevant to myocardial injury, remodeling, heart failure, or cardiomyopathy.	Non-cardiac disease without an eligible cardiac outcome; populations outside the prespecified review scope.	Experimental systems are mapped separately; they are not pooled as equivalent levels of evidence.
Disease context	Acute myocardial injury; post-injury remodeling; established heart failure; hypertrophy/pressure overload; cardiomyopathies; treatment-related cardiac injury; inflammatory cardiac disease.	Models without a relevant myocardial disease or injury context.	Disease stages are retained as distinct domains along a clinical continuum.
Intervention	Nanoparticles used as therapeutic carriers or nanomaterials with an intended intrinsic therapeutic effect.	Non-nanoparticle interventions; diagnostic-only systems without therapeutic assessment.	Carrier, payload, coating/ligand, and functional role are coded separately.
Contextual safety evidence	Nanoparticle exposure studies reporting cardiac hazard or toxicology, retained in a separate contextual stratum.	Exposure studies without relevant cardiac assessment.	These reports inform safety interpretation but do not contribute to the therapeutic evidence-density map.
Comparator	Untreated disease; vehicle/placebo; standard therapy; or the same active agent in free/conventional form, where available.	No eligible disease/control framework for the outcome being interpreted.	Only matched same-agent comparisons can establish formulation-specific advantage.
Outcomes	Cardiac function, myocardial injury, remodeling, fibrosis, structure, inflammation, oxidative stress, cell death, angiogenesis, safety, biodistribution, and clearance.	Reports without an eligible cardiac, therapeutic, safety, or fate outcome.	Evidence density is not interpreted as comparative efficacy.
Report type	Original experimental reports with sufficient information for eligibility assessment.	Reviews, editorials, protocols, abstracts without assessable data, and retracted reports.	One retracted report was removed after full-text reconciliation.

Percentages for evidence strata and model types use 157 as denominator; disease-domain and platform percentages use the 140-study main therapeutic/platform corpus

S3. Risk-of-bias tool assignment and categorization

Risk-of-bias and methodological-quality assessments were performed using design-appropriate instruments selected according to the experimental design of each study. The assignment of the primary assessment tool was mutually exclusive, such that each study contributed to only one design-specific assessment in the final risk-of-bias analysis.

Studies performed in laboratory animals were assessed using SYRCLE's risk-of-bias tool [S1]. Studies conducted exclusively in cell-based or acellular in vitro systems were assessed for methodological reliability using the in vitro ToxRTool [S2], with reliability classified according to the corresponding Klimisch categories. The cross-sectional observational human studies, Martinelli et al. (2018) [S3], was assessed using the Newcastle-Ottawa Scale adapted for cross-sectional studies [S4].

For studies combining in vivo and in vitro experiments, the assessment tool was assigned according to the pivotal experimental design underlying the primary therapeutic efficacy claims and outcomes. Because these claims and primary outcomes were derived from the in vivo experiments, mixed in vivo/in vitro studies were assessed using SYRCLE and were categorized as in vivo studies for the final risk-of-bias analysis. Their accompanying in vitro components were therefore not scored separately using ToxRTool. This approach ensured that each study contributed only once to the primary assessment and avoided double counting of mixed-design studies.

Accordingly, studies were categorized for the final assessment as follows: animal-only studies and mixed-design studies with a pivotal in vivo component contributed to the SYRCLE risk-of-bias analysis; exclusively cell-based or acellular in vitro studies contributed to the ToxRTool methodological-reliability analysis and were reported according to Klimisch reliability categories; and the cross-sectional observational human study was reported separately using the Newcastle-Ottawa Scale.

Reporting completeness of studies assigned to the in vivo category was additionally evaluated using the ARRIVE 2.0 Essential 10 criteria [S5]. The ARRIVE assessment was considered complementary to the SYRCLE risk-of-bias assessment and was used to characterize completeness of reporting rather than as an additional risk-of-bias instrument.

Reports that could not undergo a design-specific assessment because they were not original experimental studies or because the available full text did not permit a reliable assessment were classified as not assessable and were excluded from the corresponding risk-of-bias tabulation.

Supplementary Table S3. Design-specific assignment of risk-of-bias and methodological-quality assessment tools

Study design	Assessment tool	Purpose	Categorization in the final analysis
Animal / in vivo	SYRCLE risk-of-bias tool [S1]	Risk-of-bias assessment	Included in the in vivo risk-of-bias analysis
Exclusively cell-based or acellular in vitro	ToxRTool [S2]	Methodological-reliability assessment	Included in the in vitro reliability analysis; reported according to Klimisch categories
Mixed in vivo + in vitro	SYRCLE risk-of-bias tool [S1]	Risk-of-bias assessment based on the pivotal in vivo design	Counted once as an in vivo study; accompanying in vitro experiments were not separately scored with ToxRTool
Cross-sectional observational human study	Newcastle-Ottawa Scale adapted for cross-sectional studies [S4]	Methodological-quality assessment	Reported separately as an observational human study
In vivo studies - supplementary reporting assessment	ARRIVE 2.0 Essential 10 [S5]	Reporting completeness	Complementary assessment; not considered a separate risk-of-bias tool

Note. Assignment of the primary assessment tool was mutually exclusive. Each study contributed to only one design-specific primary assessment, thereby avoiding double counting. ARRIVE 2.0 was applied separately as a complementary assessment of reporting completeness in the in vivo studies.

Supplementary Results

Supplementary Tables

Supplementary Table S4. Representative studies of nanoparticle-based therapies across cardiovascular indications, with key endpoints and mechanistic features

Representative examples of the therapeutic nanoparticle platforms are presented in Table S4, which summarizes the experimental models, therapeutic cargos, intended applications, and principal mechanistic findings reported across the included studies. Despite considerable heterogeneity in nanoparticle composition and study design, several recurring mechanisms emerged, including attenuation of inflammation and oxidative stress, reduction of cardiomyocyte apoptosis and fibrosis, enhancement of angiogenesis, and preservation of ventricular function. However, direct comparisons between nanoparticle formulations and the corresponding conventional therapies remained uncommon, limiting the ability to attribute the observed therapeutic effects specifically to the nanoparticle delivery system rather than to the active therapeutic agent itself.

Supplementary Table S4 is provided as a separate file due to its size and to ensure optimal readability and accessibility of the complete study-level information.

Supplementary Table S5 Toxicity and safety-related nanoparticle studies in cardiovascular preclinical research.

Supplementary Table S5 summarizes the 17 studies retained as contextual safety/toxicology evidence, including nanoparticle type, experimental model, exposure conditions, and reported adverse cardiac or systemic effects. The findings indicate that toxicity was strongly dependent on material composition, dose, route, surface properties, and exposure duration, and should therefore be interpreted as formulation- and context-specific rather than as evidence of a uniform class effect.

Supplementary Table S5 is provided as a separate file due to its size and to ensure optimal readability and accessibility of the complete study-level information.

Supplementary Figures

Supplementary Figure S2

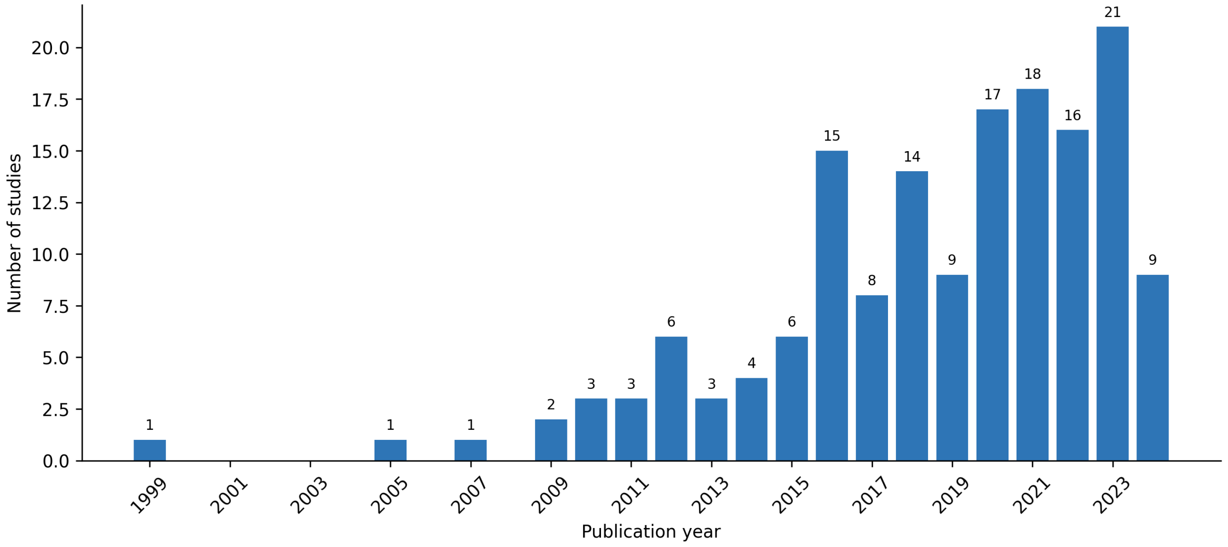

Supplementary Figure S2. Publication year of the 157 independent studies. Bars show the number of retained studies published in each year from 1999 to 2024.

The temporal distribution demonstrates a marked increase in research activity over the past decade, with most studies published after 2015 and a peak in 2023. This trend reflects the growing interest in nanomedicine as a therapeutic strategy for myocardial injury and heart failure and highlights the rapid expansion of preclinical investigations in this field.

Supplementary Figure S3

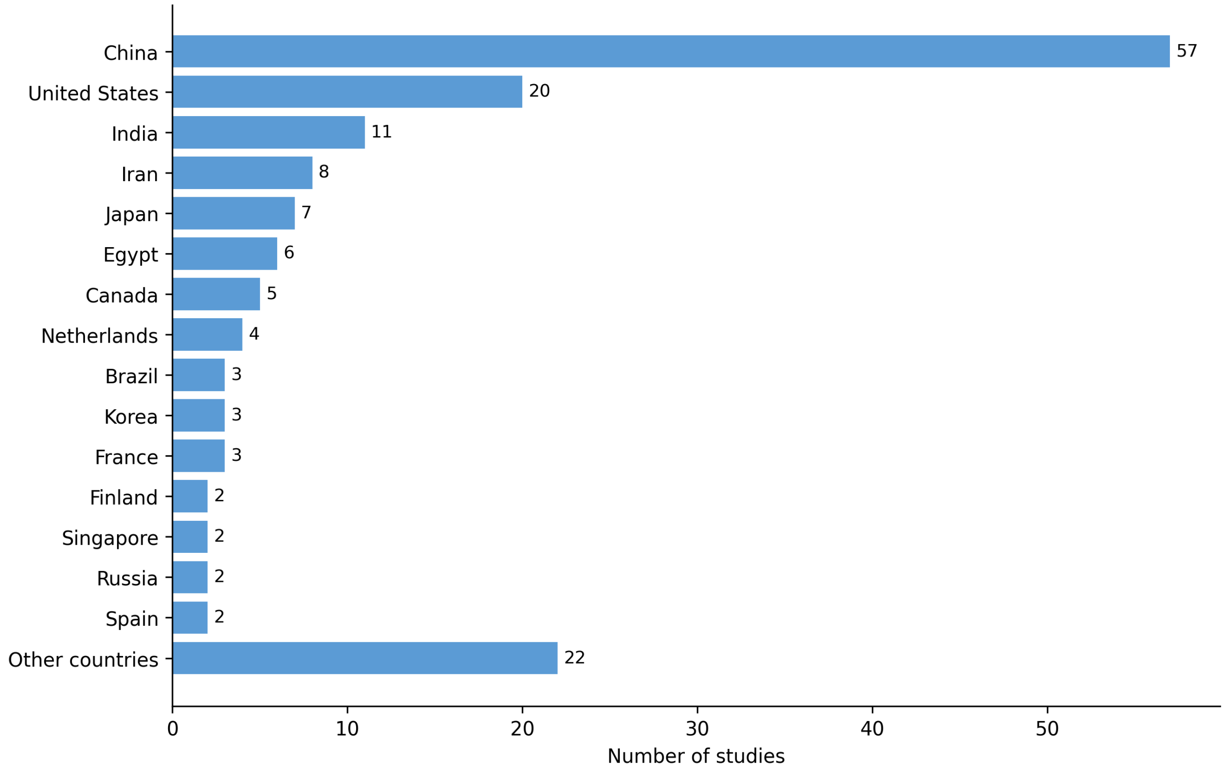

Supplementary Figure S3. Geographic distribution by country. The 15 most frequently reported countries are shown individually; remaining countries are grouped as "Other countries".

Research activity was geographically concentrated in Asia, particularly China, which accounted for the largest proportion of published studies. North America and Europe also contributed substantially, whereas studies from other regions were comparatively limited, indicating an uneven global distribution of preclinical research in cardiovascular nanomedicine.

Supplementary Figure S4

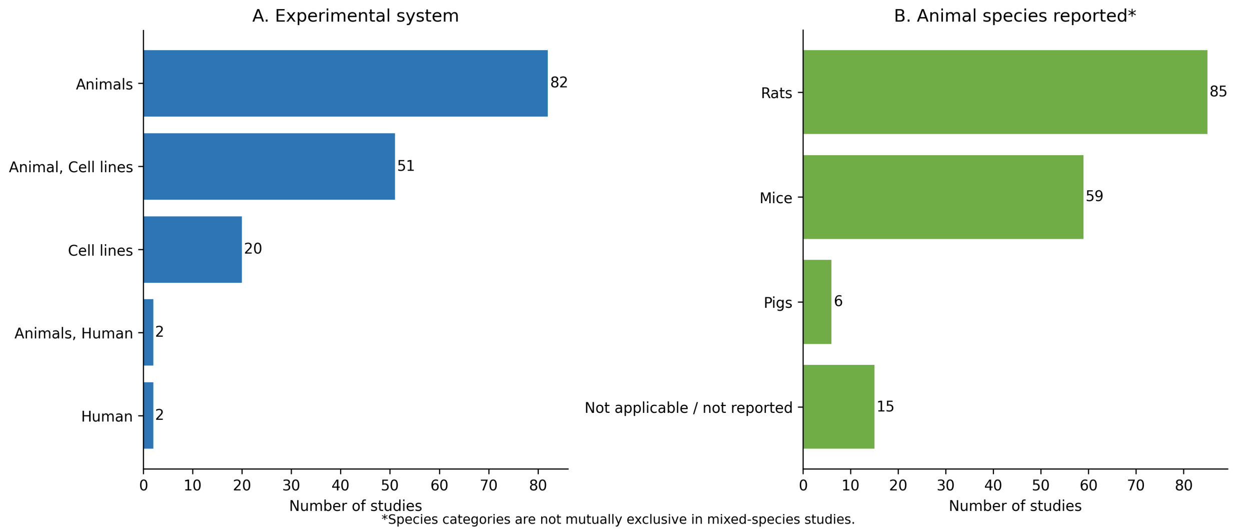

Supplementary Figure S4. Experimental systems and animal species. Panel A displays mutually exclusive experimental-system categories. Panel B displays species mentions; species counts are not mutually exclusive in mixed-species studies.

Most investigations were performed in animal models, either alone or combined with cell-based experiments, whereas exclusively human studies were rare. Rodents, particularly rats and mice, represented the predominant experimental species, reflecting their widespread use for modeling myocardial injury and evaluating nanoparticle-based therapeutic interventions.

Supplementary Figure S5

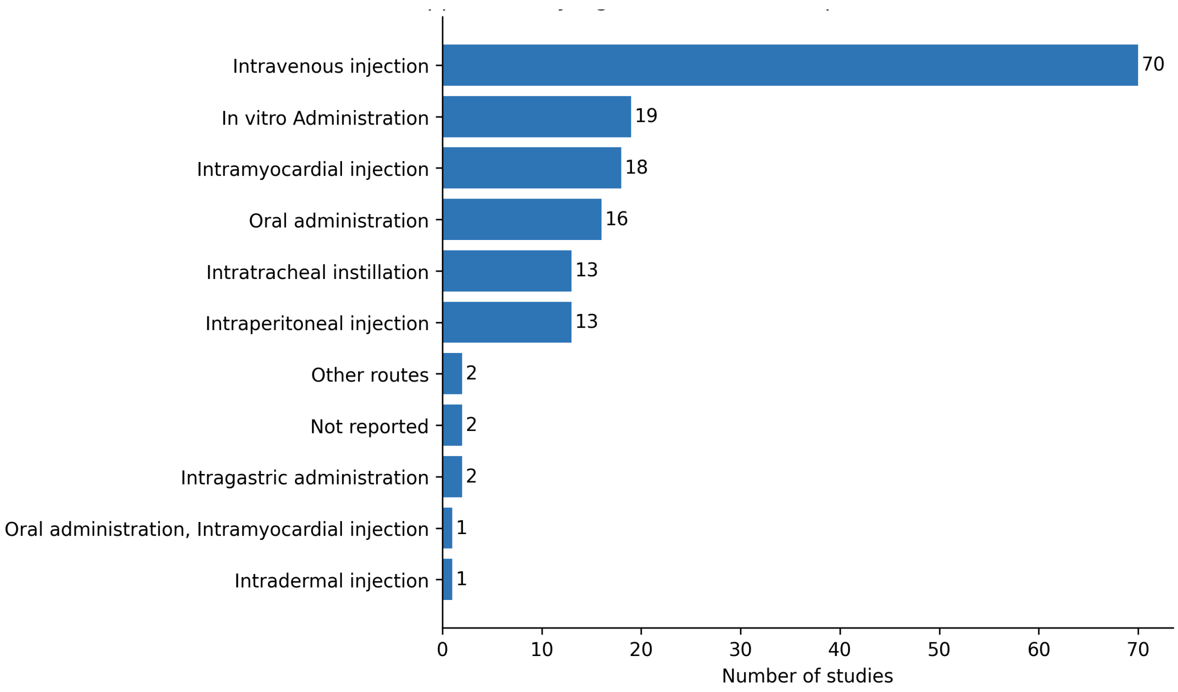

Supplementary Figure S5. Route or experimental mode of administration. Categories reproduce the harmonized route field in the final evidence database.

Intravenous administration was the most frequently reported delivery route, consistent to achieve systemic nanoparticle distribution and cardiac targeting. Other administration routes, including intramyocardial, oral, intraperitoneal, and intratracheal delivery, were less commonly employed and generally reflected disease-specific experimental designs.

Supplementary Figure S6

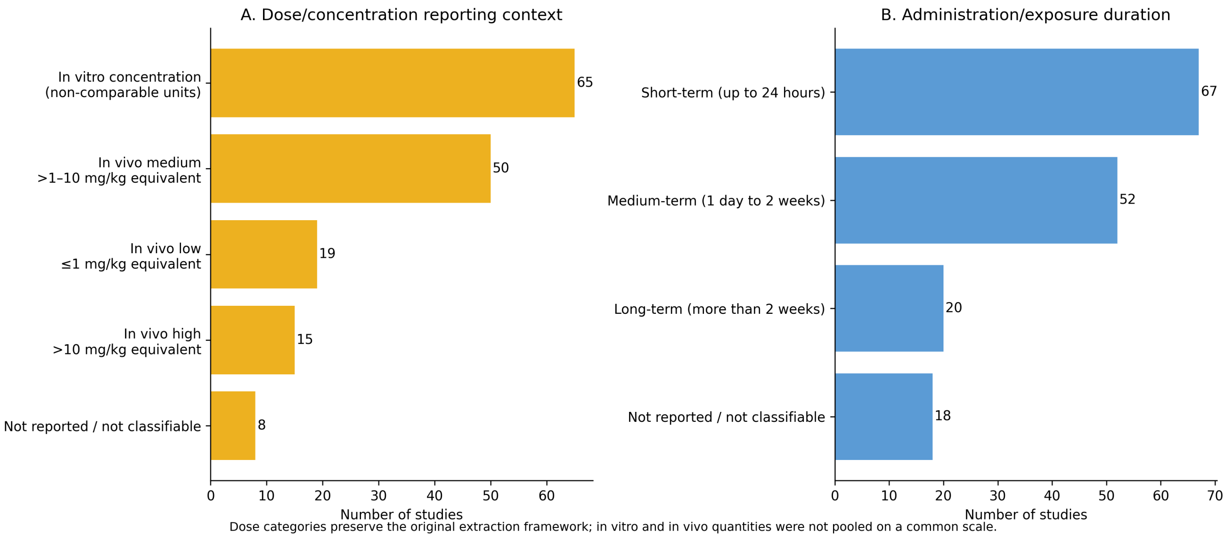

Supplementary Figure S6. Dose context and exposure duration. *In vitro* quantities and *in vivo* dose categories are displayed separately and were not pooled on a common concentration scale.

Considerable heterogeneity was observed in both dose reporting and treatment duration across the included studies. Because *in vitro* concentrations and *in vivo* doses were expressed using non-comparable units, they were analyzed separately. Most investigations evaluated short- to medium-term exposure, whereas relatively few studies assessed long-term treatment effects.

Supplementary References

S1. Hooijmans, C.R.; Rovers, M.M.; de Vries, R.B.M.; Leenaars, M.; Ritskes-Hoitinga, M.; Langendam, M.W. SYRCLE's risk of bias tool for animal studies. BMC Med. Res. Methodol. 2014, 14, 43. https://doi.org/10.1186/1471-2288-14-43.

S2. Schneider, K.; Schwarz, M.; Burkholder, I.; Kopp-Schneider, A.; Edler, L.; Kinsner-Ovaskainen, A.; Hartung, T.; Hoffmann, S. 'ToxRTool', a new tool to assess the reliability of toxicological data. Toxicol. Lett. 2009, 189, 138-144. https://doi.org/10.1016/j.toxlet.2009.05.013.

S3. Martinelli, A.; Maranhao, R.C.; Carvalho, P.O.; Freitas, F.R.; Silva, B.M.; Curiati, M.C.; Kalil Filho, R.; Pereira-Barretto, A.C. Cholesteryl ester transfer protein (CETP), HDL capacity of receiving cholesterol and status of inflammatory cytokines in patients with severe heart failure. Lipids Health Dis. 2018, 17, 242. https://doi.org/10.1186/s12944-018-0888-0.

S4. Modesti, P.A.; Reboldi, G.; Cappuccio, F.P.; Agyemang, C.; Remuzzi, G.; Rapi, S.; Perruolo, E.; Parati, G.; ESH Working Group on CV Risk in Low Resource Settings. Panethnic Differences in Blood Pressure in Europe: A Systematic Review and Meta-Analysis. PLoS ONE 2016, 11, e0147601. Supplementary S1 Text: Newcastle-Ottawa Quality Assessment Scale adapted for cross-sectional studies. https://doi.org/10.1371/journal.pone.0147601.

S5. Percie du Sert, N.; Hurst, V.; Ahluwalia, A.; Alam, S.; Avey, M.T.; Baker, M.; Browne, W.J.; Clark, A.; Cuthill, I.C.; Dirnagl, U.; et al. The ARRIVE Guidelines 2.0: Updated Guidelines for Reporting Animal Research. PLoS Biol. 2020, 18, e3000410. https://doi.org/10.1371/journal.pbio.2020.18.e3000410.
