# Supplementary material for: Nanoparticle-Based Therapies for Myocardial Injury and Heart Failure: A Systematic Review and Translational Appraisal of Preclinical Evidence
Source: Biomolecules. 2026 Aug 27;16(9):1245. doi: 10.3390/biom16091245 (PMC13604711; doi:10.3390/biom16091245)
Supplement: Supplementary file 1 [file biomolecules-16-01245-s001.zip › Table S4_Therapeutic_Nanoparticle_Platforms_renumbered.pdf]

Table S4. Representative studies of nanoparticle-based therapies across cardiovascular indications, with key endpoints and mechanistic features

Representative preclinical studies grouped by nanoparticle platform. The table summarizes the therapeutic cargo, experimental model, intended purpose, biological system, and principal mechanistic findings reported in the source dataset.

Nanoparticle platform	Nanocargo	Model	Purpose	Cell line	Animal	Key mechanistic findings
**Polymeric micelle**						
PLGA nanoparticles	CHIR99021 + FGF1	Surgically induced MI; post-infarction LV remodeling	Myocardial protection; infarct size reduction	hiPSC-CMs, HUVECs	Mouse, Pig; Human	Reduce infarct size by 20-30%, preserve LV function, decrease cardiomyocyte apoptosis, and increase angiogenesis
PLGA nanoparticles (CsA-NP)	Cyclosporine A	Myocardial ischemia-reperfusion	Enhance CsA-mediated cardioprotection at reperfusion by targeting mPTP	-	Mouse	Inhibition of mitochondrial permeability transition pore opening in cardiomyocyte, reduces infarct size and improves LV function.
RGD PEG PLA nanoparticles	miR 133	Acute MI / chronic HF model	Cardiac protection after MI	-	Male Wistar rats	Reduces infarct lesions, improves cardiac function and cardiomyocyte apoptosis via SIRT3/AMPK modulation
mPEG-PLA-TPGS nanoparticles (tanshinone IIA-NPs)	Tanshinone IIA	Myocardial ischemia-reperfusion (I/R) injury	Reduce adverse LV remodeling and improve cardiac function post-MI	-	C57BL/6 mice	NF κB pathway suppression, anti-inflammatory, limit infarct expansion and LV dilation, attenuate inflammation, apoptosis, and myocardial fibrosis
NIPAAm-MMA nanoparticles	Visnagin	Myocardial ischemia-reperfusion	Ameliorate MI/R injury and preserve cardiac function	-	Rat	Improves LV function by inducing autophagy (↑LC3) and inhibiting apoptosis (↓caspase-3/-9, TUNEL)
PEO-b-PCL nanoparticles	Enalapril maleate	Heart failure and hypertension	Sustained carrier for enalapril	-	Mice	Sustained release and good drug-retaining capability with normal weight gain and food consumption
PLGA nanoparticles	15d-PGJ2	Postinfarct ventricular dysfunction	Improve ventricular function and prevent dilation	Mesenchymal stem cells	Rats	Increased ejection fraction, reduced ventricular dilation, decreased infarct size and collagen content, increased angiogenesis and cardiomyocyte proliferation
PLGA nanoparticles	Mdivi1	Myocardial ischemia-reperfusion injury	Protect heart via inhibition of MOMP	RNVMs	Mice	Reduced infarct size and cardiomyocyte death through inhibition of mitochondrial outer membrane permeabilization
PLGA-b-PEG nanoparticles	TGF-beta3	Cardiac fibrosis	Prevent fibrosis induced by TGF-beta3	Mouse cardiac fibroblast cells	-	Reduced collagen production, SMA expression, and cell migration
PVAX nanoparticles	Vanillyl alcohol	DOX-induced cardiac toxicity	Protect heart and liver from oxidative injury	C2C12 cells	Mice	Prevented apoptosis and cardiac dysfunction; improved survival
Polydopamine nanoparticles	Polydopamine	Cardiomyocyte bioenergetics	Improve contractility and beat propagation	hiPSC-CMs	-	Enhanced mitochondrial bioenergetics and calcium cycling
Cysteine-Based Redox-Responsive Nanoparticles	PF543	Myocardial infarction	Target activated cardiac fibroblasts	Neonatal rat cardiac fibroblasts	-	Reduced fibrosis, improved cardiac function and suppressed fibroblast activation
PLGA nanoparticles	Pitavastatin	Myocardial ischemia-reperfusion injury	Reduce ischemia-reperfusion injury at reperfusion	-	Pigs	Reduced infarct size, improved LV function, and attenuated remodeling without adverse effects
Plasma polymerized nanoparticles	PDGF-AB	Injured heart	Direct delivery of growth factor therapy	Human cardiac cells and rat cells	Rats	Safe direct myocardial delivery with modest functional improvement
DAPA-MSN-CHP nanoparticles	Dapagliflozin	Heart failure after MI	Cardiac repair and regeneration	Cardiomyocytes	Mice	Reduced apoptosis and preserved viable myocardium
PLGA nanoparticles	ABT263	Ischemia-reperfusion injury / cardiac remodeling	Senolytic delivery to infarcted heart	Rat cardiomyocytes and fibroblasts	Rats	Eliminated senescent cells without systemic toxicity
Core/shell nanoparticles	VEGF	Ischemic heart regeneration	Stable localization at ischemic area	-	Rats	Improved ejection fraction, cardiac output and neovascularization
GlcNAc-decorated polyketal nanoparticles	S100A1 protein	Failing cardiac myocytes	Improve calcium handling and contractility	Primary cardiac myocytes	Rats	Reduced aberrant calcium release and restored sarcomere function
PLGA nanoparticles	miR-199a-3p	Restoring infarcted myocardium	Promote myocardial restoration	ESC-derived cardiomyocytes and endothelial cells	Rats	Improved cardiac function and angiogenesis
PLGA nanoparticles	Irbesartan	Myocardial ischemia-reperfusion injury	Antagonize monocyte-mediated inflammation	-	Mice	Reduced infarct size, inflammatory monocyte recruitment, and improved cardiac function
Dextran functionalized nanoparticles	CHIR99021 + SB431542	Cardiac fibrosis / hypertrophy	Promote direct reprogramming of cardiac fibroblasts into cardiomyocyte-like cells	Cardiac fibroblasts	Rat	Anti-fibrotic, dual inhibition of TGF β and Wnt pathways
**Dendrimers**						
PAMAM dendrimers	CTGF shRNA	Fibrotic/CTGF overexpression model	Gene knockdown of connective tissue growth factor	H9c2	Rat	Anti fibrotic via CTGF knockdown, modulation of extracellular matrix
**Liposomes**						
Nanoliposome (SMV-Lipo)	Simvastatin	Pressure overload heart failure	Prevent ISO-induced cardiac hypertrophy and fibrosis	-	BALB/c mice	Upregulation of KLF2-Foxp1 signaling, endothelial protection, inhibits cardiac hypertrophy, fibrosis, inflammation
Lipid core nanoparticles (LDE MTX)	Methotrexate	Acute myocardial infarction	Reduce infarct size and improve post-MI LV function	-	Wistar rats	Targeted anti-inflammatory delivery, plaque stabilization, improves LV systolic function, reduces infarct size, hypertrophy, fibrosis
PEGylated liposomes	Adenosine	Myocardial ischemia-reperfusion injury	Augment cardioprotection and reduce side effects	-	Rats	Reduced infarct size in a dose-dependent manner with fewer hemodynamic effects
PEG-conjugated liposomal nanoparticles	Pyr1-apelin-13	Pressure overload-induced cardiac dysfunction	Prolong apelin stability and potentiate cardioprotective effect	HEK 293 cells	Mice	Attenuated left ventricular hypertrophy and cardiac dysfunction

Nanoparticle platform	Nanocargo	Model	Purpose	Cell line	Animal	Key mechanistic findings
Lipid core nanoparticles	Methotrexate	Myocardial infarction	Improve functional and inflammatory status of heart	-	Rats	Reduced infarction size, improved LV function, reduced inflammation, oxidative stress and fibrosis
PEG-modified liposomes	Apigenin + RAGE-siRNA	Myocardial ischemia-reperfusion injury	Reduce oxidative stress and inflammation	H9C2 cells and SD rats	Rats	Reduced apoptosis, inflammation and oxidative injury via RAGE/NF-kB pathway
RGD modified solid lipid nanoparticles	Puerarin	Acute myocardial ischemia	Prolong retention and protect ischemic heart	HCM	Rats	Higher heart/plasma concentration and lowest infarct size among formulations
PLA-PEG-PLA nano-vesicles	Propolis	Heart failure	Reduce inflammatory burden and improve echo parameters	-	Rats	Decreased IL-1, IL-6, IL-17 and increased IL-10, SIRT1 and AMPK
Lipid-based cationic nanoparticles	miR-181c	Heart failure	Study mitochondrial genome and bioenergetics effects	-	Rats	Caused dysfunction in mitochondrial complex IV, increased oxidative stress and altered cardiac function
Solid lipid nanoparticles (SLNs)	Ranolazine	Hypertension / ischemia	Enhance oral bioavailability and anti-anginal/anti-ischemic efficacy of ranolazine	-	Rat	Improved pharmacokinetics, prolonged antihypertensive action
**Metal and inorganic nanoparticles**						
PEG coated gold nanoparticles	Intrinsic AuNP	Acute myocardial infarction	Improve cardiac function after myocardial infarction	-	C57BL/6 mice	Reduces post-MI fibrosis and inflammation, enhances angiogenesis
PEG-coated gold nanoparticles (AuNPs)	Intrinsic AuNP	MI / hypertrophy	Attenuate β-adrenergic receptor-mediated cardiac hypertrophy and inflammation	Neonatal rat ventricular cardiomyocytes (NRVMs)	Mouse	Anti-fibrotic, decrease β1-AR expression and downstream ERK1/2 hypertrophic signaling, limit acute cardiac hypertrophy and inflammatory response
Silver/chitosan nanoparticles	Intrinsic silver	Post-MI heart failure	Improve cardiac function and attenuate post-MI cardiac remodeling	-	Rats	Reduces infarct size and adverse remodeling, improves LV function, likely via antioxidant and anti-inflammatory effects
Cerium oxide nanoparticles	Intrinsic CeO_2	Doxorubicin induced cardiomyopathy	Prevent DOX-induced cardiac dysfunction and remodeling	-	Adult male rats (DOX-treated)	ROS scavenging, decreases oxidative stress, Restores cardiac function and contractility, modulation of autophagy and mitochondrial function
Calcium carbonate nanoparticles (ColCaNPs)	Colchicine	Acute coronary syndrome / MI	Enhance colchicine cardioprotection by targeted anti-inflammatory action	-	Rat	Anti-inflammatory, anti-pyroptotic, reduce infarct size (~45%), attenuate myocardial fibrosis
Fe2O3-DMSA nanoparticles	Intrinsic iron oxide	Ischemic injury	Cardioprotective activity and imaging	Neonatal rat cardiomyocytes	Rats and pigs	Reduced infarct size and improved cardiac function with low toxicity
Anionic maghemite nanoparticles	Maghemite	Cardiac MR imaging / graft monitoring	Enable MR detection of transplanted smooth muscle cells	Rat smooth muscle cells	Rats	Noninvasive monitoring of cell engraftment by MR imaging
Silica nanoparticles (SNP)	Adenosine	Myocardial ischemia-reperfusion injury	Targeted drug delivery into ischemic myocardium	-	Rats	More pronounced infarct size limitation and reduced hypotensive effect compared to free adenosine
Silver/chitosan nanoparticles	Ag NPs/CS-Mentha piperita	Heart failure after MI	Improve cardiac function and attenuate remodeling	-	Rats	Reduced infarct size, improved LV function, lowered LV/body and lung/body weight ratios, antioxidant effect
Selenium nanoparticles (SeNPs)	Intrinsic selenium	Isoproterenol-induced myocardial infarction	Ameliorate infarct damage and oxidative stress	-	Rats	Decreased CK-MB, AST, CRP and improved lipid/electrolyte profile; antioxidant effect
Mesoporous silica nanoparticles	Curcumin	Doxorubicin cardiotoxicity	Improve curcumin bioavailability and prevent DOX-induced myocardial toxicity	-	Rat	Enhanced curcumin bioavailability, antioxidant and anti-apoptotic effects and reduce biochemical and histopathological markers of cardiotoxicity
Curcumin CMC-peptide nanoparticles	Curcumin	Cardiac hypertrophy	Improve bioavailability and regress hypertrophy	H9c2 cells and rat cardiomyocytes	Rats	Reduced apoptotic load and improved cardiac function
**Carbon-based / theranostic nanoparticles**						
Gadolinium-doped carbon dots, PEG-QK–conjugated nanoparticles	VEGF mimetic peptide QK	Myocardial ischemia-reperfusion (I/R) injury	Theranostic MRI agent and pro-angiogenic therapy for acute MI	-	Rat	Pro-angiogenic signaling, vascular regeneration, reducing cardiac fibrosis, remodeling, and dysfunction
Melanin nanoparticles	Melanin	Sepsis-induced myocardial injury	Alleviate sepsis-induced myocardial injury via anti-ferroptosis and anti-inflammatory effects	H9c2	Mouse	Improves cardiac function and structure, reduces ROS, restores mitochondrial homeostasis, and suppresses ferroptosis and inflammatory signaling in cardiomyocyte

Abbreviations: 15d-PGJ2, 15-deoxy-Delta12,14-prostaglandin J2; AMPK, AMP-activated protein kinase; CsA, cyclosporine A; CTGF, connective tissue growth factor; DAPA, dapagliflozin; DOX, doxorubicin; ESC, embryonic stem cell; FGF1, fibroblast growth factor 1; HF, heart failure; hiPSC-CMs, human induced pluripotent stem cell-derived cardiomyocytes; HUVECs, human umbilical vein endothelial cells; I/R, ischemia-reperfusion; LV, left ventricular; MI, myocardial infarction; mPTP, mitochondrial permeability transition pore; NP, nanoparticle; PLGA, poly(lactic-co-glycolic acid); RAGE, receptor for advanced glycation end products; ROS, reactive oxygen species; SLN, solid lipid nanoparticle; TGF-beta, transforming growth factor beta; VEGF, vascular endothelial growth factor.
