# Supplementary material for: Nanoparticle-Based Therapies for Myocardial Injury and Heart Failure: A Systematic Review and Translational Appraisal of Preclinical Evidence
Source: Biomolecules. 2026 Aug 27;16(9):1245. doi: 10.3390/biom16091245 (PMC13604711; doi:10.3390/biom16091245)
Supplement: Supplementary file 1 [file biomolecules-16-01245-s001.zip › Table S5_Toxicity_Safety_17_Studies_renumbered.pdf]

Supplementary Table S5. Toxicity and safety-related nanoparticle studies in cardiovascular preclinical research.

The table summarizes the 17 contextual safety/toxicology reports retained separately from the 140-study therapeutic/platform evidence corpus. Reported effects should be interpreted as material-, dose-, route-, and exposure-specific findings rather than evidence of uniform class-wide toxicity.

No.	Nanoparticle type / platform	Disease model	Animal / cell system	Exposure route	Reported adverse effect	Proposed mechanism
1	Iron nanoparticles	Cardiomyocyte injury	HL1-NB cells (murine cardiac cells)	In vitro	Increased cell death, inflammation, and oxidative stress; slight cytotoxicity when used alone	Potentiation of 7-ketocholesterol-induced cytotoxicity through pro-oxidant and pro-inflammatory effects
2	Gold core silver nanoparticles	Cardiovascular injury / ischemia-reperfusion	Rats	Intratracheal instillation	Exacerbated ischemia-reperfusion injury and altered vascular function	Pulmonary exposure induced cardiovascular toxicity that varied by particle size and capping agent
3	Titanium dioxide nanoparticles	Cardiac inflammation and hemodynamic dysfunction	Mice	Intragastric administration	Cardiac inflammation, apoptosis, and impaired hemodynamics	Reduced ATP production and increased pro-inflammatory cytokine expression in the heart
4	PEG-coated gold nanoparticles	Cardiac hypertrophy	Mice	Intravenous injection	Reversible cardiac hypertrophy, fibrosis, inflammation, and cardiac dysfunction	Chronic exposure caused size-dependent cardiac structural remodeling
5	Silica nanoparticles	Cardiomyocyte cardiotoxicity	Rat cardiomyocytes and H9c2 cells	In vitro	Mitochondrial dysfunction, oxidative stress, impaired calcium handling, and reduced contractility	Size and dose-dependent toxicity mediated by energetic failure and calcium dysregulation
6	Silica nanoparticles and methylmercury	Cardiac toxicity	Rats and AC16 cells	Intratracheal instillation and in vitro	Severe cardiac injury, apoptosis, and mitochondrial damage	Additive or synergistic oxidative stress from co-exposure
7	Polyacrylic acid coated iron oxide nanoparticles	Cardiac toxicity	Rats, mice, human iPSC-derived cells, L929 cells, and Ana-1 cells	Intravenous injection	Cardiac toxicity requiring prevention strategy	Ion chelation and CaV1.2 perturbation; Ca^{2+} pre-chelation prevented toxicity
8	Titanium dioxide nanoparticles	Cardiac structural and functional deterioration in hypertension	Spontaneously hypertensive rats	Intratracheal instillation	Adverse cardiac remodeling and worsened hemodynamic performance	Subchronic inflammatory, oxidative, and profibrotic responses
9	Silica nanoparticles	Myocardial contractile dysfunction	Rats and AC16 cells	Intratracheal instillation	Myocardial contractile dysfunction and inflammatory injury	JNK/TF/PAR1 mediated inflammatory and oxidative signaling
10	Amorphous silica nanoparticles	Cardiac dysfunction	Rat heart and human cardiomyocytes	In vitro	Impaired cardiac function, mitochondrial injury, and ATP depletion	ROS generation, mitochondrial permeability transition pore opening, and reduced ATP synthesis
11	Silver nanoparticles	Myocardiocyte structural alterations	Rats	Intratracheal instillation	Cardiac dysfunction and ultrastructural abnormalities	Disrupted mitochondrial dynamics and blocked mitochondrial autophagy
12	Gold nanoparticles	Cardiomyocyte apoptosis / acute myocardial injury	Rats	Intratracheal instillation	Cardiomyocyte apoptosis and histopathological cardiac injury	Mitochondrial pathway activation and dose-dependent toxicity
13	Hydroxyapatite nanoparticles	Potential cardiac risk	Wistar male rats	Oral administration	Cardiac toxicity and oxidative inflammatory injury	Hydroxyapatite-induced cardiac damage; chitosan and curcumin nanoformulations were protective
14	Amorphous silica nanoparticles	Systemic inflammation and fibrosis	ApoE-/- mice	Intratracheal instillation	Fibrosis in the heart and other organs	Mast cell-mediated release of inflammatory mediators, including IL-4, IL-13, GM-CSF, and TARC
15	Silica nanoparticles	Cardiac injury and dysfunction	ApoE-/- mice and AC16 cells	Intratracheal instillation	Cardiac impairment, dysfunction, remodeling, and apoptosis	ROS/Ca^{2+}/CaMKII signaling and calcium overload
16	Amorphous silica nanoparticles	Ferroptosis-associated cardiomyocyte injury	Rats and AC16 cells	Intratracheal instillation	Myocardial injury and cardiomyocyte damage	miR-125b-2-3p/HO-1 signaling, iron overload, and lipid peroxidation
17	Carbon black nanoparticles	Myocardial cell damage	Mice and HL-1 cardiomyocytes	Intratracheal instillation	Myocardial damage, apoptosis, and autophagy defects	ROS-dependent mitochondrial dysfunction, impaired mitophagy, and inflammatory activation

Abbreviations: ATP, adenosine triphosphate; CaMKII, calcium/calmodulin-dependent protein kinase II; GM-CSF, granulocyte-macrophage colony-stimulating factor; HO-1, heme oxygenase-1; iPSC, induced pluripotent stem cell; JNK, c-Jun N-terminal kinase; PAR1, protease-activated receptor 1; PEG, polyethylene glycol; ROS, reactive oxygen species; TARC, thymus- and activation-regulated chemokine; TF, tissue factor.
